# EGDB: A comprehensive multi‐omics database for energy grasses and the epigenomic atlas of pearl millet

**DOI:** 10.1002/imt2.263

**Published:** 2024-12-28

**Authors:** Lin Luo, Dongmei Lin, Jinhui Li, Hao Chen, Qi Qu, Lin Zhang, Yuan Luo, Jiaming Chen, Dingkun Jiang, Peitao Lü, Wenjun Zhu, Hui Lin, Ensi Shao, Haidong Yan, Yarong Jin, Guodong Lu, Zhanxi Lin, LuLu Xun, Fangjie Zhu, Linkai Huang, Jiajing Xiao

**Affiliations:** ^1^ College of Life Science, National Engineering Research Center of JUNCAO, Fujian Provincial Key Laboratory of Haixia Applied Plant Systems Biology, Haixia Institute of Science and Technology Fujian Agriculture and Forestry University Fuzhou China; ^2^ Juncao Science and Ecology College Fujian Agriculture and Forestry University Fuzhou China; ^3^ Department of Biology Saint Louis University Saint Louis Missouri USA; ^4^ National Key Laboratory for Tropical Crop Breeding, Institute of Tropical Bioscience and Biotechnology Chinese Academy of Tropical Agricultural Sciences Sanya China; ^5^ College of Grassland Science and Technology Sichuan Agricultural University Chengdu China; ^6^ Xi'an Botanical Garden, Institute of Botany Xi'an China

## Abstract

Given the key role of energy grasses in biomass energy, electricity, biofuels, and carbon sequestration, the Energy Grass Omics Database (EGDB) integrates germplasm data with genomics, transcriptomics, epigenomics, and phenomics data to support functional genomic research on diverse energy grass species. EGDB also currently supplies the largest epigenetic data set of energy grasses: a high‐resolution chromatin modification, chromatin accessibility, and gene expression landscape of pearl millet to provide insights into regulatory traits essential for sustainable energy production.

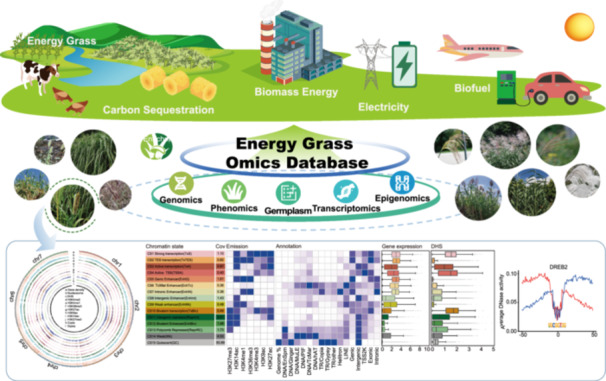

To the Editor,

With the increasing demand for sustainable energy solutions, energy grasses have emerged as vital crops due to their high yields [1]. These grasses also play a crucial role in carbon sequestration and capture, contributing to the reduction of greenhouse gas emissions [2, 3]. Additionally, energy grasses enhance biodiversity and improve soil health, fostering ecological balance. Integrating these grasses into sustainable practices not only contributes to climate change mitigation but also supports rural economic revitalization [4]. Recent advancements in high‐throughput sequencing have generated large‐scale biological datasets across multiple omics layers for energy grasses [5, 6]. However, no comprehensive database is currently available for energy grasses. To bridge this gap, we developed the Energy Grass Database (EGDB, https://engrass.juncaodb.cn/), integrating a multi‐omics database from eleven key energy grass species. EGDB integrates germplasm data with genomics, transcriptomics, epigenomics, and phenomics, covering species from genera such as *Cenchrus*, *Miscanthus*, and *Arundo*.

In the past few years, epigenomic maps have been constructed for many economic plants such as *Oryza sativa*, *Brassica napus*, and *Triticum aestivum* [7, 8, 9]. These efforts have generated an abundance of data that not only assisted the annotation of the noncoding genome but also unraveled the fundamental mechanisms of plant transcriptional regulation. While genomic and transcriptomic data have been generated for multiple energy grasses, their epigenetic datasets remained scarce [10]. Therefore, focusing on the energy grasses pearl millet (excellent drought tolerance, extensively grown in arid and semi‐arid regions) [11], we constructed a comprehensive epigenomic map utilizing MNase‐seq, ChIP‐seq, and DNase‐seq, generating 163 Gb sequencing data to illustrate nine epigenetic features. These datasets were also included in EGDB. The epigenetic datasets will facilitate the systematic annotation of the potential noncoding genome, such as the annotation of chromatin states and *cis*‐regulatory elements, and provide insights into the transcriptional regulatory mechanisms [12].

Altogether, EGDB serves as a centralized platform, providing researchers with the integration of data resources and toolkits to explore key genes, regulatory networks, and epigenetic modifications that are relevant to the future trait engineering of bioenergy grasses.

## RESULTS AND DISCUSSION

### Overview of EGDB

**Figure 1:**
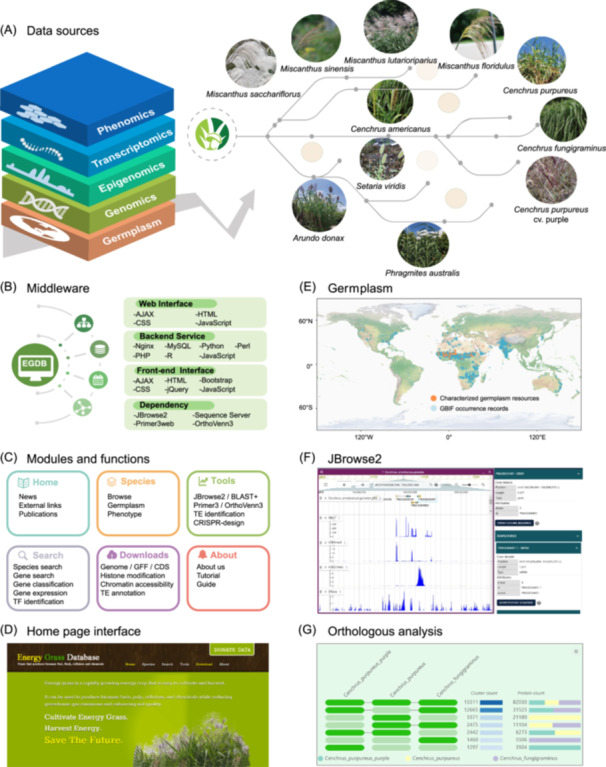
Energy grass database construction and overview and key functionalities. (A) Data source integration: The database integrates multi‐omics data across several layers, including epigenomics, phenomics, germplasm, transcriptomics, and genomics, to provide comprehensive insights into energy grass species. Key species represented include *Miscanthus lutarioriparius*, *Miscanthus sacchariflorus*, *Setaria viridis*, *Cenchrus purpureus*, *Arundo donax*, and *Phragmites australis*. (B) Middleware infrastructure: The architecture utilizes a layered middleware design for efficient data processing and management, with backend services implemented in Python, PHP, and Perl, while the frontend interface employs AJAX, HTML, CSS, and JavaScript. Various dependency modules like JBrowse2, Sequence Server, and OrthoVenn3 are included for seamless data visualization and analysis. (C) Modules and functions: The database offers a comprehensive set of features, including species browsing, germplasm information, BLAST tools, CRISPR analysis, gene search functionalities, and download options for genomic resources such as GFF and gene annotation files. (D) Homepage interface: The homepage emphasizes energy grass research applications for sustainable energy production and biomass optimization. It encourages contributions and offers a user‐friendly interface for accessing diverse data resources. (E) Germplasm distribution: Global distribution pattern of pearl millet (*C. americanus*) germplasm diversity. The map illustrates the spatial distribution of characterized germplasm accessions (orange dots) and GBIF (Global Biodiversity Information Facility) occurrence records (blue dots). The extensive geographical distribution spanning diverse ecological zones demonstrates the species' broad environmental adaptability and phenotypic plasticity, underscoring its significant potential as a bioenergy feedstock. (F) JBrowse2 visualization: Visualization of genomic features for pearl millet (*C. americanus*) in the JBrowse2 genome browser. This example demonstrates the integration and visualization of multi‐omics datasets, including gene models (*PMA3G04401*), RNA‐seq expression data, histone modifications (H3K4me3 and H3K27me3), and DNase‐seq chromatin accessibility signals across a selected region (chr3: 164,928,166−165,063,568). The energy grass database supports the visualization of transcriptomic data across different tissues and experimental conditions, as well as genomic data for various energy grass species. (G) Orthologous analysis: The analysis demonstrates the distribution of orthologous gene clusters across *C. purpureus* cv. purple, *C. fungigraminus*, and *C. purpureus*. Green bars indicate the presence of orthologous clusters within each species, while black connecting lines denote shared clusters. Cluster count represents the total number of orthologous clusters, and Protein count reflects the total associated proteins. For instance, *C. purpureus* cv. purple contains 15,511 clusters comprising 82,030 proteins. This functionality in EGDB allows the exploration of species‐specific and shared gene clusters for evolutionary and functional insights.

Energy grasses are essential for advancing sustainable bioenergy solutions due to their high biomass yields and their adaptability to marginal lands. Because no comprehensive database is currently available for energy grasses. Here, we constructed the Energy Grass DataBase (EGDB), a comprehensive, multidimensional platform for energy grasses of the Poaceae family. EGDB includes eleven species: *Cenchrus fungigraminus* (JUJUNCAO), *Cenchrus purpureus* (Napier grass), *Cenchrus purpureus* cv. purple (Purple Napier grass), *Miscanthus sinensis* (Chinese silvergrass), *Miscanthus floridulus* (Awn grass), *Miscanthus sacchariflorus* (Amur silvergrass), *Miscanthus lutarioriparius* (Fat‐stemmed miscanthus), *Arundo donax* (Giant reed), *Phragmites australis* (Common reed), *Setaria viridis* (Green foxtail), and *Pennisetum glaucum* syn. *Cenchrus americanus* (Pearl millet) (Figure 1A, Figure S1). The database contains 19.64 Gb of genomic sequences, encompassing a total of 539,598 genes, with 342,300 of these genes functionally annotated. Furthermore, a total of 28,897 transcription factors were systemically identified, providing a complete transcription factor (TF) annotation for energy grasses. From these grass species, we also identified 17,143,635 transposable elements (TEs) loci of multiple TE families. Additionally, EGDB integrates 267 gene expression profiles from 16 tissue types, enabling in‐depth exploration of tissue‐specific and conditional‐specific expression patterns. These genetic and transcriptomic data resources set up a foundation to illustrate genome structures and gene functions of energy grasses.

EGDB is built on a modular middleware architecture that effectively integrates modern frontend technologies, including AJAX, HTML (https://html.spec.whatwg.org/), and CSS (https://www.w3.org/Style/CSS/), with a robust backend powered by Nginx (https://nginx.org/en/download.html) and MySQL (https://dev.mysql.com/downloads/) (Figure 1B). To enhance data processing and analysis, the platform utilizes programming languages, including Python (https://www.python.org/downloads/), R (https://cran.r-project.org/), and Perl (https://www.perl.org/get.html), enabling efficient management of large‐scale omics datasets. Additionally, EGDB integrates essential tools, including JBrowse2 (https://jbrowse.org/jb2/download/) for advanced genome visualization and Sequence Server (https://sequenceserver.com/) for high‐throughput sequence retrieval. This structure significantly enhanced EGDB's capability to support complex genomic research. EGDB is organized into six primary functional modules: Home, Species, Tools, Search, Downloads, and About (Figure 1C).

The Home module serves as the main gateway, including sections such as news, external links, and publications to keep users informed about recent advancements, relevant resources, and key studies (Figure 1D). The Species module allows users to explore the comprehensive collection of energy grass species, including detailed germplasm information and phenotype data (Figure 1E, Figure S2). This module also provides the ecological distribution, stress resistance traits, and biomass production characteristics for each species. Moreover, users can also access germplasm resources to gain insights into the genetic background, propagation materials, and phenotype‐phenotype relationships of each species (Figure S3). The Search module offers an interface for exploring the database. Users can search for species, genes, genomic locations, agronomic traits, gene classification, gene expression, and TFs (Figure S3). The Tools module offers a suite of bioinformatics tools designed for advanced genomic research. Core tools include JBrowse2 and BLAST+ for genome visualization and sequence analysis (Figure 1F), Primer3 for efficient primer design (Figure S4), and OrthoVenn3 for ortholog analysis (Figure 1G). These tools enable users to perform gene search, functional annotation, sequence alignment, and gene family analysis. EGDB also provides the collection of TEs identified genome‐wide (Figure S4) and guide RNA designs for each gene that can readily be applied for CRISPR editing. The Downloads module offers access to an extensive collection of genomic and epigenomic data resources, including genome sequences, GFF annotations, CDS sequences, histone modification, chromatin accessibility, and TE annotations.

Therefore, by integrating extensive multi‐omics datasets and bioinformatics tools, EGDB offers a valuable resource for dissecting gene functions and regulatory elements of energy grasses.

### Epigenetic features of pearl millet

**Figure 2:**
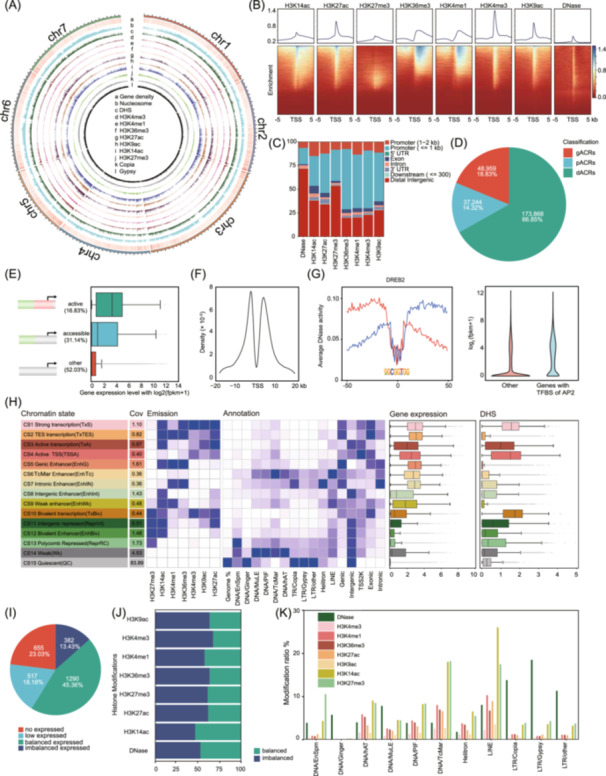
The epigenomic map of pearl millet helped to unveil the regulatory mechanism for gene expression. (A) Genome‐wide characterization of the chromosomal distribution of epigenomic features in pearl millet. The outermost circle represents the seven chromosomes at a 100‐kb resolution. The second circle shows gene density. Moving inward from the outermost to innermost circles: the distribution of nucleosome positions; DNase I hypersensitive site (DHS); seven histone modification marks (H3K4me3, H3K36me3, H3k27ac, H3k9ac, H3k14ac, and H3k27me3); and the distribution of LTR retrotransposons elements (Copia and Gypsy). (B) Heatmaps for seven histone modifications and open chromatin signals around the TSSs of genes. (C) Bar chart showing genomic distribution across those epigenomic marks in leaf tissue, including promoter (1−2 kb), promoter (≤1 kb), 5′UTR, 3′UTR, exon, intron, downstream (≤300 bp), and distal intergenic. (D) Genome‐wide distribution of three types of accessible chromatin regions‐genic ACRs (gACRs), promoter ACRs (pACRs), and distal ACRs (dACRs) ‐based on their proximity to genes. (E) The expression level of genes with active promoters (active), accessible promoters (accessible), and non‐accessible promoters (other). (F) Distribution of the distance of intergenic ACRs to nearest genes. (G) DNA footprints analysis suggested the significantly enriched AP2 transcription factors (left) and their effect on gene expression (right). (H) The chromatin landscape of pearl millet is characterized by a 15‐state model. From left to right, the features for each chromatin state (CS) include the definition and abbreviations of each CS, coverage of each CS, composition of histone modifications, enrichments of genomic annotations, expression level of genes associated with each CS, and the corresponding DHS levels. (I) The imbalanced expression pattern for most expressed duplicated paralogues. (J) The imbalanced epigenomic features contributed to the imbalanced expressed duplicated paralogues. (K) Histone modification level in the major TE families.

For energy grasses, while genomic and transcriptomic data are available, epigenetic datasets remain scarce. To supplement EGDB with epigenetic data, we also constructed a comprehensive epigenomic map for pearl millet. Pearl millet is extensively grown in arid and semi‐arid regions (about 30 million hectares) [11]. While used as an energy grass, the cereal of pearl millet also feeds more than 90 million people worldwide [13]. Combining MNase‐seq, ChIP‐seq, DNase‐seq, and RNA‐seq, we generated 163 Gb of epigenetic sequence data (Figure 2, Figure S5, and Table S1). Through systematic annotation of the noncoding genome, we defined 15 chromatin states and potential *cis*‐regulatory elements, including 27,396 accessible promoters and 173,868 distal regulatory elements (potential enhancers). The epigenetic map reveals that the active histone marks are enriched either in promoter regions (H3K4me3, H3K27ac, H3K14ac, and H3K9ac) or in the genic regions (H3K4me1 and H3K36me3) (Figure 2A−B, S6−8, and Table S2). In pearl millet, 71.70% of DNase I hypersensitive sites (DHSs) are located in the intergenic region (Figures 2C), and 53.11% of the intergenic open chromatin regions overlap with TEs, particularly with Gypsy elements, which are present in 23.18% of the intergenic DHSs. For the repressive histone mark, 53.74% of H3K27me3 peaks are found in the intergenic region, which may contribute to the genome compaction and repression of TEs.

Based on DHSs, 255,629 accessible chromatin regions (ACRs, Figure 2D) were identified. These ACRs were classified into genic ACRs (gACRs, overlapping ≥1 bp with annotated genes), proximal ACRs (pACRs, within the 2 kb flanking region of genes, but not overlapping the genes), and distal ACRs (dACRs, >2 kb from their nearest genes). Active promoters (featured with DHSs and H3K4me3) were associated with genes with the highest expression level, and the inaccessible (“other”) promoters were associated with the lowest expression (Figure 2E, Figure S9). A total of 42,311 active enhancers were defined, with 13,673 located in intergenic regions. Most intergenic enhancers are located within a 10 kb range flanking the nearest genes (Figure 2F), positioning them within proximity for transcriptional regulation. Accordingly, genes with nearby enhancers exhibit higher transcriptional activity compared to genes lacking enhancers (Figure S9B). In addition, 68,408 transcription factor binding sites (TFBSs) were spotted in the DHS regions. From the DHSs of leaves, motif discovery analysis shows that Apetala2 (AP2) TFs are significantly enriched (Figure S9C), implicating the activity of AP2 TFs in leaf development. Consistently, target genes of AP2 TFs exhibited higher expression levels. The targets of AP2 targets are involved in processes such as translation, RNA biosynthetic process, RNA metabolic process, and regulation of DNA‐templated transcription with protein binding functions (Figure 2G, S10). The genome‐wide distribution of nucleosomes was also profiled to characterize nucleosome occupancy patterns near transcription start sites (TSS) and transcription end sites (TES) (Figure S11). Histone modification levels across eight nucleosome clusters were also analyzed (Figure S12). We found that genes with high nucleosome occupancy across the gene body (Cluster 1) display elevated H3K27me3 levels and are associated with low expression, indicating that nucleosome occupancy and repressive histone modifications can work in concert to silence gene expression. In contrast, Cluster 7, characterized by low nucleosome occupancy, shows high levels of active modifications in promoter and gene body regions. Accordingly, genes in Cluster 7 are associated with the highest expression levels.

Based on the genome‐wide epigenomic features, 15 chromatin states (CSs) were defined in pearl millet (Figure 2H, S12, and S13). Active states (CS1−4) are associated with high transcriptional activity and active histone modifications. Enhancers‐related states (CS5−9) represent active or poised enhancers located in genic or intergenic regions. Repressive states (CS10−13) featured H3K27me3 in combination with H3K14ac and H3K27ac (Figure S14). Inactive states (CS14−15) lack histone modifications and are enriched with DNA TEs or LTR retrotransposon elements. Among these chromatin stats, we found two new chromatin states (CS11 and CS12) that are intermediate between the repressive and poised states. These intermediate states feature the co‐occupancy of H3K27me3 and H3K14ac, with or without H3K27ac, and the detailed biological information for those combinations required for further investigation.

Dosage imbalance was extensively observed and is considered to have adaptive photosynthesis value in evolution [14, 15]. Among the 2844 duplicated paralogues, we assessed how their dosage is influenced by epigenomic features and found that 77.15% of them displayed imbalanced expression. This is in agreement with their imbalanced epigenomic marks (Figure 2I−J). The active epigenomic mark H3K4me3 shows the highest imbalance, generally enriched in the paralogue with the higher expression (Figure S15A−B). For instance, pearl millet has two copies of phosphoenolpyruvate carboxykinase (PEPCK), a core enzyme of the C4 pathway. Among them, *PEPCK1* (*PMA1G01509*) is associated with elevated H3K4me3 levels, consistent with its higher expression (Figure S15C). Conversely, the repressive mark H3K27me3 also shows a significant imbalance but is more enriched in the paralogue with lower expression. Furthermore, because pearl millet features C4 photosynthetic pathway and a high yield, we also systematically visualized the expression and histone modification of genes involved in the “Photosynthesis” and “Photosynthesis_C4 photosynthesis” pathways in MAPMAN (Figure S16). The genome of pearl millet comprises a substantial proportion (69.36%) of TEs. To explore their regulatory potential, we analyzed the epigenomic marks associated with TEs (Figure 2K). Many LTR retrotransposon elements were found to be associated with high chromatin accessibility, with 18.51% of Gypsy and 13.82% of Copia elements located in DHS regions, suggesting that their expansion may also contribute to distal regulatory elements for gene expression. In contrast, DNA TEs are more frequently marked by histone modifications, particularly H3K27me3.

Overall, the epigenomic map of pearl millet enables a comprehensive annotation of the noncoding regions of its genome and provides valuable insights into the molecular mechanisms underlying the transcriptional regulation of pearl millet.

## CONCLUSION

By integrating comprehensive genomic, epigenomic, transcriptomic, and phenotypic information, together with the bioinformatics tools, EGDB offers a multifunctional platform for future scientific and translational research of energy grasses and fosters sustainable agriculture and renewable energy development.

## METHODS

The platform integrates multi‐omics datasets from eleven energy grasses, encompassing genomics, epigenomics, transcriptomics, and phenomics data to support functional genomic research across diverse energy grass species. The user interface provides an intuitive experience for accessing these data resources via Vue3. The data in EGDB is managed by a MySQL server, which can manage data efficiently. Nine epigenetic features of pearl millet were profiled using MNase‐seq, DNase‐seq, and ChIP‐seq. A 15‐state chromatin state model for pearl millet was generated using ChromHMM, and the epigenetic features of the energy grasses can be visualized using JBrowse2. Detailed information is available in the supplementary material.

## AUTHOR CONTRIBUTIONS

**Lin Luo:** Conceptualization; writing—original draft; project administration; writing—review and editing. **Dongmei Lin:** Data curation; methodology; writing—original draft. **Jinhui Li:** Investigation; software; writing—review and editing. **Hao Chen:** Methodology; formal analysis; writing—review and editing. **Qi Qu:** Data curation; software. **Lin Zhang:** Data curation; validation. **Yuan Luo:** Validation; methodology. **Jiaming Chen:** Formal analysis; visualization. **Dingkun Jiang:** Investigation; data curation. **Peitao Lü:** Methodology; validation. **Wenjun Zhu:** Methodology; validation. **Hui Lin:** Data curation; validation. **Ensi Shao:** Methodology; validation. **Haidong Yan:** Supervision; project administration.**Yarong Jin:** Formal analysis; visualization. **Guodong Lu:** Methodology; resources. **Zhanxi Lin:** Resources; Data curation. **LuLu Xun:** Investigation; resources. **Fangjie Zhu:** Supervision; funding acquisition; writing—review and editing. **Linkai Huang:** Supervision; resources; writing—review and editing. **Jiajing Xiao:** Conceptualization; supervision; writing—review and editing.

## CONFLICT OF INTEREST STATEMENT

The authors declare no conflict of interest.

## ETHICS STATEMENT

No animals or humans were involved in this study.

## Supporting information


**Figure S1:** Global distribution and vegetation cover types of three energy grass species.
**Figure S2:** Content display of the search module in the Energy Grass Database (EGDB).
**Figure S3:** Gene expression and phenotype analysis functions in the EGDB.
**Figure S4:** The tools of the EGDB.
**Figure S5:** Epigenomic marks featured by ChIP‐seq.
**Figure S6:** Distribution of modification level of seven histone modifications and DHS across all genes in the genome of pearl millet.
**Figure S7:** Clustering analysis reveals that genes associated with histone modifications exhibit varying levels of expression.
**Figure S8:** GO enrichment analysis was performed on the genes in the eight clusters.
**Figure S9:** The architecture of open chromatin region in pearl millet.
**Figure S10:** GO enrichment analysis of genes targeted by AP2 transcription factors.
**Figure S11:** The landscape of nucleosome position and occupancy in pearl millet.
**Figure S12:** The epigenetic and transcriptomic patterns of genes based on their corresponding nucleosome occupancy.
**Figure S13:** Chromatin State Distribution around TSS and TES.
**Figure S14:** Examples of genes affected by two newly identified chromatin states.
**Figure S15:** Imbalanced epigenetic modifications contribute to differential gene expression.
**Figure S16:** Epigenetic and transcriptomic profiles in pearl millet.

**Table S1:** Summary of 21 sequencing libraries of pearl millet from our study.
**Table S2:** Distribution of histone modification marks and open chromatin signal in pearl millet genome.

## Data Availability

All data generated and analyzed in this study are available in the NCBI Sequence Read Archive under PRJNA749489 (https://www.ncbi.nlm.nih.gov/bioproject/?term=PRJNA749489) and in the China National GeneBank DataBase (CNGBdb) at https://bigd.big.ac.cn/gsa/browse/CRA016351, or included in Supplementary materials. The datasets and scripts have been deposited on the EGDB website (https://engrass.juncaodb.cn/) and GitHub (https://github.com/ashelylinluo/EGDB). Supplementary materials (methods, figures, tables, graphical abstract, and updated materials) may be found in the online DOI or iMeta Science http://www.imeta.science/.
