# Supplementary material for: EGDB: A comprehensive multi‐omics database for energy grasses and the epigenomic atlas of pearl millet
Source: Imeta. 2024 Dec 28;4(1):e263. doi: 10.1002/imt2.263 (PMC11865331; doi:10.1002/imt2.263)
Supplement: Supplementary file 1 — Figure S1: Global distribution and vegetation cover types of three energy grass species. Figure S2: Content display of the search module in the Energy Grass Database (EGDB). Figure S3: Gene expression and phenotype analysis functions in the EGDB. Figure S4: The tools of the EGDB. Figure S5: Epigenomic marks featured by ChIP‐seq. Figure S6: Distribution of modification level of seven histone modifications and DHS across all genes in the genome of pearl millet. Figure S7: Clustering analysis reveals that genes associated with histone modifications exhibit varying levels of expression. Figure S8: GO enrichment analysis was performed on the genes in the eight clusters. Figure S9: The architecture of open chromatin region in pearl millet. Figure S10: GO enrichment analysis of genes targeted by AP2 transcription factors. Figure S11: The landscape of nucleosome position and occupancy in pearl millet. Figure S12: The epigenetic and transcriptomic patterns of genes based on their corresponding nucleosome occupancy. Figure S13: Chromatin State Distribution around TSS and TES. Figure S14: Examples of genes affected by two newly identified chromatin states. Figure S15: Imbalanced epigenetic modifications contribute to differential gene expression. Figure S16: Epigenetic and transcriptomic profiles in pearl millet. [file IMT2-4-e263-s001.docx]

**Supporting information to**

**EGDB: A Comprehensive Multi-Omics Database for Energy Grasses and the Epigenomic Atlas of Pearl Millet**

**Running title**: EGDB: multi-omics database for energy grasses and pearl millet epigenomic atlas

Lin Luo^1#^, Dongmei Lin^2#^, Jinhui Li^3#^, Hao Chen^2#^, Qi Qu^1^, Lin Zhang^1^, Yuan Luo^2^, Jiaming Chen^2^, Dingkun Jiang^1^, Peitao Lü^5^, Wenjun Zhu^1^, Hui Lin^2^, Ensi Shao^2^, Haidong Yan^4^, Yarong Jin^4^, Guodong Lu^1^, Zhanxi Lin^2^, LuLu Xun^6^, Fangjie Zhu^1*^, Linkai Huang^4*^, Jiajing Xiao^1*^

^1^College of Life Science, National Engineering Research Center of JUNCAO, Fujian Provincial Key Laboratory of Haixia Applied Plant Systems Biology, Haixia Institute of Science and Technology, Fujian Agriculture and Forestry University, Fuzhou 350002, China

^2^Juncao Science and Ecology College, Fujian Agriculture and Forestry University, Fuzhou 350002, China

^3^Department of Biology, Saint Louis University, Saint Louis, MO 63103, USA

^4^College of Grassland Science and Technology, Sichuan Agricultural University, Chengdu 611130, China

^5^National Key Laboratory for Tropical Crop Breeding, Institute of Tropical Bioscience and Biotechnology, Chinese Academy of Tropical Agricultural Sciences, Sanya 572024, China

^6^Xi'an Botanical Garden, Institute of Botany, Shaanxi Province, Xi'an 710000, China

#These authors contributed equally: Lin Luo, Dongmei Lin, Jinhui Li, Hao Chen

*Correspondence:

[jjxiao@fafu.edu.cn](mailto:jjxiao@fafu.edu.cn%20) (Jiajing Xiao),

[fjzhu@fafu.edu.cn](mailto:fjzhu@fafu.edu.cn) (Fangjie Zhu),

[huanglinkai@sicau.edu.cn](mailto:huanglinkai@sicau.edu.cn) (Linkai Huang)

**METHODS**

**Data collection**

The Energy Grass DataBase (EGDB) integrates extensive datasets from reputable resources to provide comprehensive multi-omics data for energy grasses. Genomic data were sourced from major repositories, including the National Center for Biotechnology Information (NCBI, <https://www.ncbi.nlm.nih.gov/>), the National Genomics Data Center (CNGB, <https://db.cngb.org/>), and the U.S. Department of Energy Joint Genome Institute (JGI, <https://genome.jgi.doe.gov/>). Genome assemblies for energy grass species, such as *Cenchrus fungigraminus*, *Cenchrus purpureus*, and *Miscanthus lutarioriparius*, were collected from published studies [1−9]. Transcriptomic data, primarily from the NCBI Sequence Read Archive (SRA), serve as the foundation for the database, featuring 267 RNA-seq datasets spanning more than 15 plant tissues, including roots, leaves, axillary buds, stem sections, inflorescences, growing stems, leaf axils, spikes, and buds. This data supports studies on gene expression across developmental stages and environmental stress conditions. Epigenomic data, including histone modification (ChIP-seq) and chromatin accessibility (ATAC-seq) datasets for energy grasses, were obtained from the NCBI Gene Expression Omnibus (GEO). Key agronomic trait data were extracted from published studies and integrated into the EGDB [10], covering traits such as dry biomass yield (Yld), culm dry weight (CmDW), total number of culms (TCmN), culms per area (Cm/A), first heading date (HD1), and other related characteristics. The literature data on energy grasses were systematically collected before October 1st, 2024, by searching the keyword “energy grass” on PubMed (<https://pubmed.ncbi.nlm.nih.gov/>), yielding a total of 12,225 results.

**Plant materials and growth conditions**

Seeds of *Cenchrus americanus* (*C. americanus*) cultivar ‘Tifleaf 3’ were obtained from Sichuan Agricultural University and planted in the cultivation base of the National Engineering Research Centre of JUNCAO Technology [11]. After drying at 37 °C, seeds were sealed in 10 mL centrifuge tubes and stored in a 4 °C refrigerator. When ready for germination, seeds were covered with filter paper and moistened with distilled water in Petri dishes. Subsequently, the seeds were cultivated in the dark at 25 °C for 12 h in a biochemical incubator. Once germinated, the seeds were transferred to pots (10 cm × 10 cm) filled with vermiculite. The plants were grown in an artificial climate chamber (28 °C day/25 °C night, an illumination intensity of 6000 lux, relative humidity of 70%−80%, and light-dark cycle of 14 h: 10 h). Because pearl millet utilizes the C4 photosynthetic pathway, we focused on the leaf tissue as a representative for epigenetic mapping. New leaf tissue was collected 15 days later for genome-wide sequencing of DNase I hypersensitive sites (DNase-seq), histone modification ChIP-seq, and RNA-seq analyses.

**ChIP-seq library preparation**

Based on our group’s previous research results, we adopted their experimental methods and sample processing procedures for ChIP-seq with some modifications [12]. Briefly, leaf tissue (third leaf at the fully expanded stage, approximately 0.1 g per replicate) was collected and ground into powder using liquid nitrogen. The cell membranes were lysed using RIPA lysis buffer (without sucrose), with dithiothreitol (DTT) and protease Inhibitor Cocktail (100 ×, cat. no.C0101; Beijing Lablead Biotech Co., Ltd.) replacing *β*-mercaptoethanol and PMSF [13]. The ground plant tissue powder was washed three times with pre-cooled RIPA buffer at 4 °C, centrifuging at 800 g for 2 min each time to remove impurities, and the supernatant was discarded after each wash. The pellet was resuspended in MNase buffer (140 mM KCl, 20 mM Tris–HCl pH 7.5, 3 mM CaCl_2_, 300 mM sucrose) and digested with MNase enzyme (37.5 U/μL, cat. no. M0247S; New England Biolabs, Inc) at 37 ℃ for 8 min with gentle shaking. MNase was used in ChIP-seq to digest chromatin into nucleosome-level DNA fragments (~150 bp). This step improves the resolution and does not require specific equipment such as the sonicator. The reactions were terminated by adding EDTA (final concentration 23.8 mM, pH 8.0). The cell lysates were centrifuged at 16,874  rpm for 2 min, and the supernatants were collected and incubated with Pierce Protein A/G magnetic beads (Thermo Scientific, cat no.88803) pre-washed with binding buffer, along with the specific antibody.

ChIP was performed using the following antibodies: H3K27me3 (ABclonal, A16199), H3K4me1 (ABclonal, A2355), H3K27ac (ABclonal, A7253), H3K14ac (Millipore, 07-353), H3K9ac (Millipore, 07-352), H3K36me3 (Abcam, ab9050), and H3K4me3 (Millipore, 07-473). The incubation was performed at 4 °C for 2 h to ensure sufficient binding of the antibody to the target protein. Following the manufacturer’s recommendations, VAHTS Universal Adapter Ligation Module for Illumina V2 (N203-02, N204-02, Vazyme Biotech Co., Ltd., China) was used to prepare sequencing libraries, and index codes were added to attribute sequences to samples. Libraries were quantified using a Qubit 4.0 fluorometer (Invitrogen, Thermo Fisher Scientific, cat. no. Q33238) and validated by qPCR. Libraries were diluted at appropriate concentrations for sequencing and then sequenced on the Illumina NovaSeq 6000 platform (Annoroad Gene Technology Co., Ltd, China). Two biologically independent samples were prepared.

**DNase-seq library preparation**

Plant samples were lysed with a modified RIPA buffer (as used in ChIP-seq) and centrifuged. The supernatant was discarded and the cell nuclei were resuspended in DNase buffer (5 mM MgCl_2_, 10 mM KCl, 1 mM CaCl_2_,100 mM NaCl, 10 mM Tris–HCl pH 7.5, and 300 mM sucrose) with protease inhibitors [14]. The pelleted nuclei were divided into 200 μL plastic centrifugal tubes on ice, followed by the addition of DNase I Enzyme (M0303L; New England Biolabs) with different concentrations (0.002 to 0.5 U). After an 8-minute incubation at 37°C, the reaction was terminated with a buffer containing 0.25M EDTA and 3.125% SDS. Proteinase K solution was added to each microtube (final concentration, 0.25 μg/μL) and incubated at 55 °C for 10 min. Afterwards, the samples were centrifuged and the supernatant was collected. DNA fragments of different lengths were separated using Agarose gel (3%) to obtain 50−200  bp fragments, which were purified using the HiPure Gel Pure DNA Mini Kit (Magen, China) [19]. The protocol for DNase-seq library preparation for Illumina sequencing followed the same procedure as described previously. Two biologically independent samples were prepared.

**RNA-seq library preparation**

Fifteen-day-old seedlings were harvested and placed in a 2 mL centrifuge tube with 4-mm stainless steel beads. The collected samples (0.1 g) were homogenized in liquid nitrogen with a high-throughput tissue grinder (Shanghai Jingxin Industrial Development Co., Ltd, China) at 50 Hz for 90 s, repeated twice. The mixed samples were sent to Novogene (Novogene, China) with dry ice for RNA isolation and library preparation. After assessing RNA quality, eukaryotic mRNA was enriched using magnetic beads with Oligo(dT). Fragmentation buffer was added to shear the mRNA into short fragments. First-strand cDNA was synthesized using random hexamer primers and the fragmented mRNA as a template. Subsequently, buffer, dNTPs, DNA polymerase I, and RNase H were added to synthesize the second strand of cDNA. The double-stranded cDNA was purified using AMPure XP beads (Beckman Coulter Co., Ltd., cat. no. A63882, USA). End repair, A-tailing, and adapter ligation were performed on the purified cDNA, followed by size selection using AMPure XP beads. PCR amplification was conducted, and the PCR products were purified with AMPure XP beads to obtain the final sequencing library. RNA-seq data were obtained for three biological replicates, with 6 Gb of sequencing data generated per sample.

**ChIP and DNase sequencing data analysis**

Raw paired-end reads were trimmed using fastp (version 0.23.4) [15] with parameters “-W 5 -M 20 -5 -3 -l 50”. High-quality reads from ChIP-seq libraries were aligned to the PI537069 genome using bowtie2 (version 2.5.1) [16]. Reads that were mapped to multiple locations were removed. Mapped reads with a MAPQ quality score below 20 and PCR duplicates were filtered using SAMTools (version 1.17) to ensure high-quality aligned data [17]. The Pearson correlation between two biological replicates was evaluated with deepTools (version 3.5.4) using 10-kb bin size [18]. For analysis of H3K4me3, H3K14ac, H3K9ac, and H3K27ac, narrow-peak calling settings were used in MACS2 (version 2.2.9.1) [19]. For analysis of H3K36me2, H3K4me1, and H3K27me3, the broad-peak mode was used in MACS2 with “--broad-cutoff 0.1 -q 0.05”. DHSs were identified with the DNase-seq dataset using MACS2.

**RNA-seq analysis**

The adapter sequences and low-quality reads were filtered using fastp (version 0.23.4) with parameters “-W 5 -M 20 -5 -3 -l 50”. The cleaned reads were mapped to PI537069 genome using Hisat2 (version 2.2.1) and only properly paired reads were kept for further analysis [20]. Gene expression was quantified using HTseq-count (version 2.0.3) [21], and the expression level of each gene was then normalized to Fragments Per Kilobase Million (FPKM) based on library size and gene length. Expressed genes were defined as those with FPKM > 1, and non-expressed genes were classified as those with FPMK = 0. Genes with FPKM values between 0 and 1 were categorized as lowly expressed. Genes with a 1-bp overlap with annotated transposon elements (TEs) were classified TE-derived genes. The statistical differences in gene expression between each comparison group were assessed using Student's *t*-test. GO enrichment analyses were performed using clusterProfiler [22].

**Characterization of chromatin states**

ChromHMM (version 1.23) [23], a multivariate Hidden Markov Model, was used for unsupervised segmentation of the PI537069 genome into 15 states based on the intensity of the seven histone modifications (H3K4me3, H3K4me3, H3K36me3, H3K27ac, H3K9ac, H3K14ac, and H3K27me3) in non-overlapping 200-bp bins. The open chromatin intensity in each chromatin state region was evaluated based on DNase-seq dataset and the expression level of each chromatin state region was evaluated by the expression level of overlapping genes. The 15-state model was the common model for animals and plants with enough efficient information.

**Epigenome analysis around genes**

Considering the histone characteristics of genes, the levels of seven histone modifications and open chromatin signals around genes, including the promoter region (2000 bp upstream) and the gene body, were evaluated and normalized to FPKM. Unsupervised clustering revealed 8 typical categories of combinations around genes using log2 transformed FPKM dataset in R (version 4.4.0) and the signal enrichment was plotted using computeMatrix, plotProfile, and plotHeatmap from deepTools (version 3.5.4) surrounding TSS sites or gene body regions.

**Detection of transcription factor-binding motifs**

To detect enriched transcription factor-binding motifs in these open chromatin regions, we first detected DHS footprints in DHS peaks using HINT (Hmm-based IdeNtification of Transcription factor footprints, version 1.0.2) [24]. A total of 68,408 DHS footprints were identified across the pearl millet genome. Homer was used to identify enriched motifs in those regions with a plant database [25]. The motifs were then scanned using the Find Individual Motif Occurrences (FIMO) program of the MEME software toolkit (version 5.5.4) [26]. The number of motifs in the promoter and enhancer-like regions was normalized against the total number of scanned regions. The target genes of AP2 superfamily were identified based on the location of motifs from AP2 superfamily.

**Homolog analysis of genes**

Homology analyses for genes annotated in the genomes of pearl millet and the two subgenomes of *C. fungigraminus* (JUJUNCAO) [1] were performed using OrthoFinder (version 2.5.5) [27] with default settings. This analysis identified 18,402 single-copy genes, 2844 duplicated paralogues, and 1771 groups with more than two gene copies in pearl millet. Genes overlapping with TEs were classified into TE-inserted genes (TE genes), while the other genes were classified into TE-free genes (non-TE genes). The expression difference between TE genes and non-TE genes was tested using Student's *t*-test, while the expression difference of TE-inserted gene and TE-free gene in duplicated orthologues were evaluated using a Wilcoxon test in R (version 4.4.0). Gene expression and epigenomic intensity of genes involved in “Photosynthesis” and “Photosynthesis_C4photosynthesis” pathways are examined using MAPMAN (version 3.6.0) [28] and the heatmaps were plotted in R. Expression bias among duplicated paralogues was assessed, identifying biased-expressed genes with at least a two-fold higher expression level than their counterpart.

**Visualization of germplasm sample distribution for energy grasses**

The EGDB provides a visualization function for germplasm distribution, showcasing the geographic distribution and ecological adaptability of energy grasses on a global scale. To enhance the database, we combined species distribution records from the Global Biodiversity Information Facility (GBIF, <https://www.gbif.org>) with existing germplasm data to analyze the distribution patterns of *C. americanus*, *Miscanthus sinensis* (*M. sinensis*), *Phragmites australis* (*P. australis*), and *Arundo donax* (*A. donax*). Distribution points of *C. americanus*, derived from GBIF records and characterized germplasm resources, were directly mapped onto the Natural Earth 50M Raster Dataset (NE2 50M SR, <https://www.naturalearthdata.com/downloads/50m-raster-data/50m-natural-earth-2/>). This visualization highlights global distribution patterns, offering an intuitive reference to understand the spatial density and geographic spread of pearl millet germplasm.

For *M. sinensis*, *P. australis*, and *A. donax*, GBIF distribution records were combined with the Global Land Surface Satellite - Global Land Cover (GLASS-GLC) dataset [29]. Spatial aggregation quantified regional distribution density, while distribution points were overlaid with land cover types (e.g., cropland, forest, and grassland) to examine the ecological adaptability of these species in diverse ecosystems. This functionality provides strong technical support for studying the geographic distribution and ecological adaptability of energy grass germplasm. It allows users to explore the resource potential and global applicability of energy grasses, offering a scientific foundation for their sustainable development and utilization.

**Identification of transcription factor families**

Transcription factors in the genome of energy grass were systematically identified using iTAK (version 1.8) [30], applying default parameters to ensure standardized classification of transcription factors and transcriptional regulators through integrated protein domain analysis. Gene Ontology (GO) annotations were subsequently conducted using clusterProfiler [22] to provide comprehensive insights into gene functions across biological processes (BP), molecular functions (MF), and cellular components (CC).

**Identification of transposable elements**

The Transposable Elements (TEs) in each energy grass were annotated using EDTA (version 2.0.1) with default parameters [31]. Unclassified long terminal repeats (LTRs) were further annotated with DeepTE (version 12.14) [32] The final GFF file and results are accessible through JBrowse2 for easy visualization and analysis.

**Design of CRISPR/Cas9 sgRNAs for energy grass genomes**

We designed single-guide RNAs (sgRNAs) for CRISPR/Cas9 editing in energy grass genomes using CRISPR-Local, a tool developed for precise sgRNA prediction and off-target analysis [33]. The sgRNA design process was conducted using default parameters to ensure optimal targeting efficiency and specificity.

**Statistical analysis**

For genes with active promoters (indicated by both DHS and H3K4me3, labeled as 'active'), accessible promoters (indicated only by DHS, labeled as 'accessible'), or genes without DHS (labeled as 'other'), statistical differences in gene expression between comparison groups were evaluated using a Student's *t*-test. The same method was used to assess statistical differences for genes targeted by Apetala2 (AP2) transcription factors (TFs) versus those not targeted by AP2 TFs. For duplicated paralogs, statistical differences were evaluated based on the expression level of genes between two groups: one set of genes with TE insertion and the other without TE, as well as genes with high H3K4me3 levels (labeled as ‘high’) versus those with low H3K4me3 levels (labeled as ‘low’). A Wilcoxon test was applied for these comparisons. All statistical analyses were conducted in R (version 4.4.0).

**REFERENCES**

1. Zheng, Huakun, Baiyu Wang, Xiuting Hua, Ruiting Gao, Yuhao Wang, Zixin Zhang, Yixing Zhang, et al. 2023. “A near-complete genome assembly of the allotetrapolyploid *Cenchrus fungigraminus* (JUJUNCAO) provides insights into its evolution and C4 photosynthesis.” *Plant Communications* 4:100633. https://doi.org/10.1016/j.xplc.2023.100633

2. De Vega, Jose, Iain Donnison, Sarah Dyer, and Kerrie Farrar. 2021. “Draft genome assembly of the biofuel grass crop *Miscanthus sacchariflorus*.” *F1000Research* 10:29. https://doi.org/10.12688/f1000research.44714.1

3. Mamidi, Sujan, Adam Healey, Pu Huang, Jane Grimwood, Jerry Jenkins, Kerrie Barry, Avinash Sreedasyam, et al. 2020. “A genome resource for green millet *Setaria viridis* enables discovery of agronomically valuable loci.” *Nature Biotechnology* 38:1203−1210. https://doi.org/10.1038/s41587-020-0681-2

4. Mitros, Therese, Adam M. Session, Brandon T. James, Guohong Albert Wu, Mohammad B. Belaffif, Lindsay V. Clark, Shengqiang Shu, et al. 2020. “Genome biology of the paleotetraploid perennial biomass crop *Miscanthus*.” *Nature Communications* 11:5442. https://doi.org/10.1038/s41467-020-18923-6

5. Miao, Jiashun, Qi Feng, Yan Li, Qiang Zhao, Congcong Zhou, Hengyun Lu, Danlin Fan, et al. 2021. “Chromosome-scale assembly and analysis of biomass crop *Miscanthus lutarioriparius* genome.” *Nature Communications* 12:2458. https://doi.org/10.1038/s41467-021-22738-4

6. Zhang, Guobin, Chunxia Ge, Pingping Xu, Shukai Wang, Senan Cheng, Yanbin Han, Yancui Wang, et al. 2021. “The reference genome of *Miscanthus floridulus* illuminates the evolution of Saccharinae.” *Nature Plants* 7:608−618. https://doi.org/10.1038/s41477-021-00908-y

7. Li, Ao, Ai Liu, Xin Du, Jinyuan Chen, Mou Yin, Hongyin Hu, Nawal Shrestha, et al. 2020. “A chromosome-scale genome assembly of a diploid alfalfa, the progenitor of autotetraploid alfalfa.” *Horticulture Research* 7:194. https://doi.org/10.1038/s41438-020-00417-7

8. Oh, Dongha, Kurt P. Kowalski, Quynh N. Quach, Chathura Wijesinghege, Philippa Tanford, Maheshi Dassanayake, and Keith Clay. 2022. “Novel genome characteristics contribute to the invasiveness of *Phragmites australis* (common reed).” *Molecular Ecology* 31:1142−1159. https://doi.org/10.1111/mec.16293

9. Yan, Qi, Fan Wu, Pan Xu, Zongyi Sun, Jie Li, Lijuan Gao, Liyan Lu, et al. 2021. “The elephant grass (*Cenchrus purpureus*) genome provides insights into anthocyanidin accumulation and fast growth.” *Molecular Ecology Resources* 21:526−542. https://doi.org/10.1111/1755-0998.13271

10. Njuguna, Joyce N., Lindsay V. Clark, Alexander E. Lipka, Kossonou G. Anzoua, Larisa Bagmet, Pavel Chebukin, Maria S. Dwiyanti, et al. 2023. “Genome-wide association and genomic prediction for yield and component traits of *Miscanthus sacchariflorus*.” *GCB Bioenergy* 15:1355−1372. https://doi.org/10.1111/gcbb.13097

11. Sun, Min, Haidong Yan, Aling Zhang, Yarong Jin, Chuang Lin, Lin Luo, Bingchao Wu, et al. 2023. “Milletdb: a multi-omics database to accelerate the research of functional genomics and molecular breeding of millets.” *Plant Biotechnology Journal* 21:2348−2357. https://doi.org/10.1111/pbi.14136

12. Wen, Chenjin, Zhen Yuan, Xiaotian Zhang, Hao Chen, Lin Luo, Wanying Li, Tian Li, et al. 2023. “Sea-ATI unravels novel vocabularies of plant active cistrome.” *Nucleic Acids Research* 51:11568−11583. https://doi.org/10.1093/nar/gkad853

13. Tu, Xiaoyu, María Katherine Mejía-Guerra, Jose A. Valdes Franco, David Tzeng, Poyu Chu, Wei Shen, Yingying Wei, et al. 2020. “Reconstructing the maize leaf regulatory network using ChIP-seq data of 104 transcription factors.” *Nature Communications* 11:5089. https://doi.org/10.1038/s41467-020-18832-8

14. Nepon-Sixt, Brook S., Victoria L. Bryant, and Mark G. Alexandrow. 2019. “Myc-driven chromatin accessibility regulates Cdc45 assembly into CMG helicases.” *Communications Biology* 2:110. https://doi.org/10.1038/s42003-019-0353-2

15. Chen, Shifu, Yanqing Zhou, Yaru Chen, and Jia Gu. 2018. “fastp: an ultra-fast all-in-one FASTQ preprocessor.” *Bioinformatics* 34:i884−i890. https://doi.org/10.1093/bioinformatics/bty560

16. Langmead, Ben, and Steven L. Salzberg. 2012. “Fast gapped-read alignment with Bowtie 2.” *Nature Methods* 9:357−359. https://doi.org/10.1038/nmeth.1923

17. Li, Heng, Bob Handsaker, Alec Wysoker, Tim Fennell, Jue Ruan, Nils Homer, Gabor Marth, Goncalo Abecasis, Richard Durbin, and 1000 Genome Project Data Processing Subgroup. 2009. “The sequence alignment/map format and SAMtools.” *Bioinformatics* 25:2078−2079. https://doi.org/10.1093/bioinformatics/btp352

18. Ramírez, Fidel, Friederike Dündar, Sarah Diehl, Björn A. Grüning, and Thomas Manke. 2014. “deepTools: a flexible platform for exploring deep-sequencing data.” *Nucleic Acids Research* 42:W187−191. https://doi.org/10.1093/nar/gku365

19. Zhang, Yong, Tao Liu, Clifford A. Meyer, Jérôme Eeckhoute, David S. Johnson, Bradley E. Bernstein, Chad Nusbaum, et al. 2008. “Model-based analysis of ChIP-Seq (MACS).” *Genome Biology* 9:R137. https://doi.org/10.1186/gb-2008-9-9-r137

20. Kim, Daehwan, Joseph M Paggi, Chanhee Park, Christopher Bennett, and Steven L. Salzberg. 2019. “Graph-based genome alignment and genotyping with HISAT2 and HISAT-genotype.” *Nature Biotechnology* 37: https://doi.org/10.1038/s41587-019-0201-4

21. Anders, Simon, Paul Theodor Pyl, and Wolfgang Huber. 2015. “HTSeq--a python framework to work with high-throughput sequencing data.” *Bioinformatics* 31:166−169. https://doi.org/10.1093/bioinformatics/btu638

22. Yu, Guangchuang, Ligen Wang, Yanyan Han, Qingyu He. 2012. “ClusterProfiler: an R package for comparing biological themes among gene clusters.” *Omics: a Journal of Integrative Biology* 16: https://doi.org/10.1089/omi.2011.0118

23. Ernst, Jason, and Manolis Kellis. 2012. “ChromHMM: automating chromatin-state discovery and characterization.” *Nature Methods* 9:215−216. https://doi.org/10.1038/nmeth.1906

24. Gusmao, Eduardo G., Manuel Allhoff, Martin Zenke, and Ivan G. Costa. 2016. “Analysis of computational footprinting methods for DNase sequencing experiments.” *Nature Methods* 13:303−309. https://doi.org/10.1038/nmeth.3772

25. Duttke, Sascha H., Max W. Chang, Sven Heinz, and Christopher Benner. 2019. “Identification and dynamic quantification of regulatory elements using total RNA.” *Genome Research* 29:1836−1846. https://doi.org/10.1101/gr.253492.119

26. Bailey, Timothy L., Mikael Boden, Fabian A. Buske, Martin Frith, Charles E. Grant, Luca Clementi, Jingyuan Ren, Wilfred W. Li, and William S. Noble. 2009. “MEME SUITE: tools for motif discovery and searching.” *Nucleic Acids Research* 37:W202−208. https://doi.org/10.1093/nar/gkp335

27. Emms, David M., and Steven Kelly. 2019. “OrthoFinder: phylogenetic orthology inference for comparative genomics.” *Genome Biology* 20:238. https://doi.org/10.1186/s13059-019-1832-y

28. Thimm, Oliver, Oliver Bläsing, Yves Gibon, Axel Nagel, Svenja Meyer, Peter Krüger, Joachim Selbig, Lukas A. Müller, Seung Y. Rhee, and Mark Stitt. 2004. “MAPMAN: a user-driven tool to display genomics data sets onto diagrams of metabolic pathways and other biological processes.” *The Plant Journal* 37:914−939. https://doi.org/10.1111/j.1365-313x.2004.02016.x

29. Liu, Han, Peng Gong, Jie Wang, Nicholas Clinton, Yuqi Bai, and Shunlin Liang. 2020. “Annual dynamics of global land cover and its long-term changes from 1982 to 2015.” *Earth System Science Data* 12:1217−1243. https://doi.org/10.5194/essd-12-1217-2020

30. Zheng, Yi, Chen Jiao, Honghe Sun, Hernan G. Rosli, Marina A. Pombo, Peifen Zhang, Michael Banf, et al. 2016. “iTAK: a program for genome-wide prediction and classification of plant transcription factors, transcriptional regulators, and protein kinases.” *Molecular Plant* 9:1667−1670. https://doi.org/10.1016/j.molp.2016.09.014

31. Ou, Shujun, Weija Su, Yi Liao, Kapeel Chougule, Jireh R. A. Agda, Adam J. Hellinga, Carlos Santiago Blanco Lugo, et al. 2019. “Benchmarking transposable element annotation methods for creation of a streamlined, comprehensive pipeline.” *Genome Biology* 20:275. https://doi.org/10.1186/s13059-019-1905-y

32. Yan, Haidong, Aureliano Bombarely, and Song Li. 2020. “DeepTE: a computational method for *de novo* classification of transposons with convolutional neural network.” *Bioinformatics* 36:4269−4275. https://doi.org/10.1093/bioinformatics/btaa519

33. Sun, Jiamin, Hao Liu, Jianxiao Liu, Shikun Cheng, Yong Peng, Qinghua Zhang, Jianbing Yan, Haijun Liu, and Lingling Chen. 2019. “CRISPR-Local: a local single-guide RNA (sgRNA) design tool for non-reference plant genomes.” *Bioinformatics* 35:2501−2503. https://doi.org/10.1093/bioinformatics/bty970


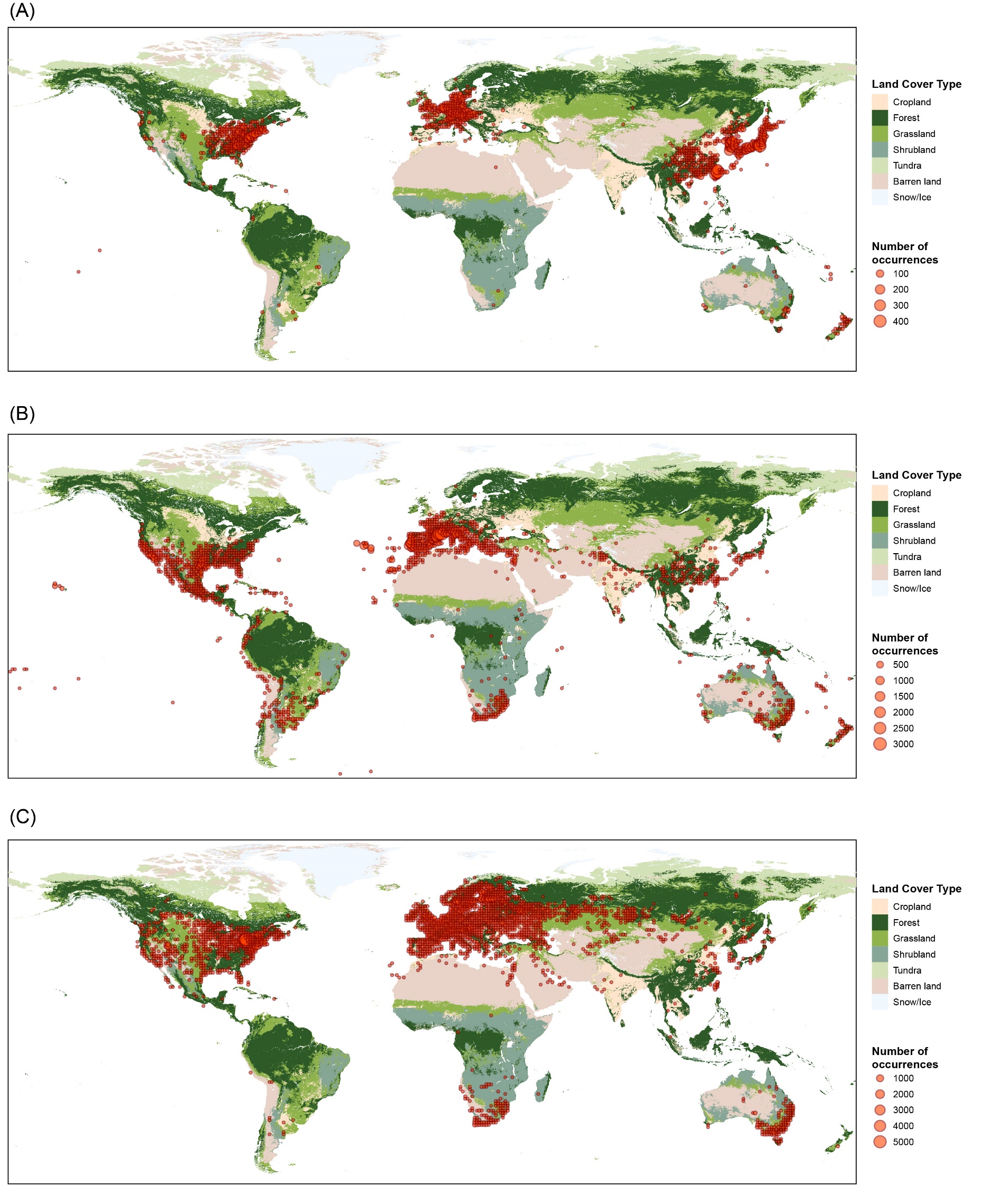


**Figure S1 Global distribution and vegetation cover types of three energy grass species.** The background map represents the 2015 GLASS-GLC land cover classification, with land cover types categorized as cropland, forest, grassland, shrubland, tundra, barren land, and snow/ice. Distribution records for each species are depicted as red circles, with circle size proportional to occurrence density aggregated to a 1° grid resolution. (A) Global distribution of *M. sinensis*. (B) Global distribution of *A. donax*. (C) Global distribution of *P. australis*. Data sources: GLASS-GLC and GBIF occurrence datasets, filtered and aggregated for spatial analysis.


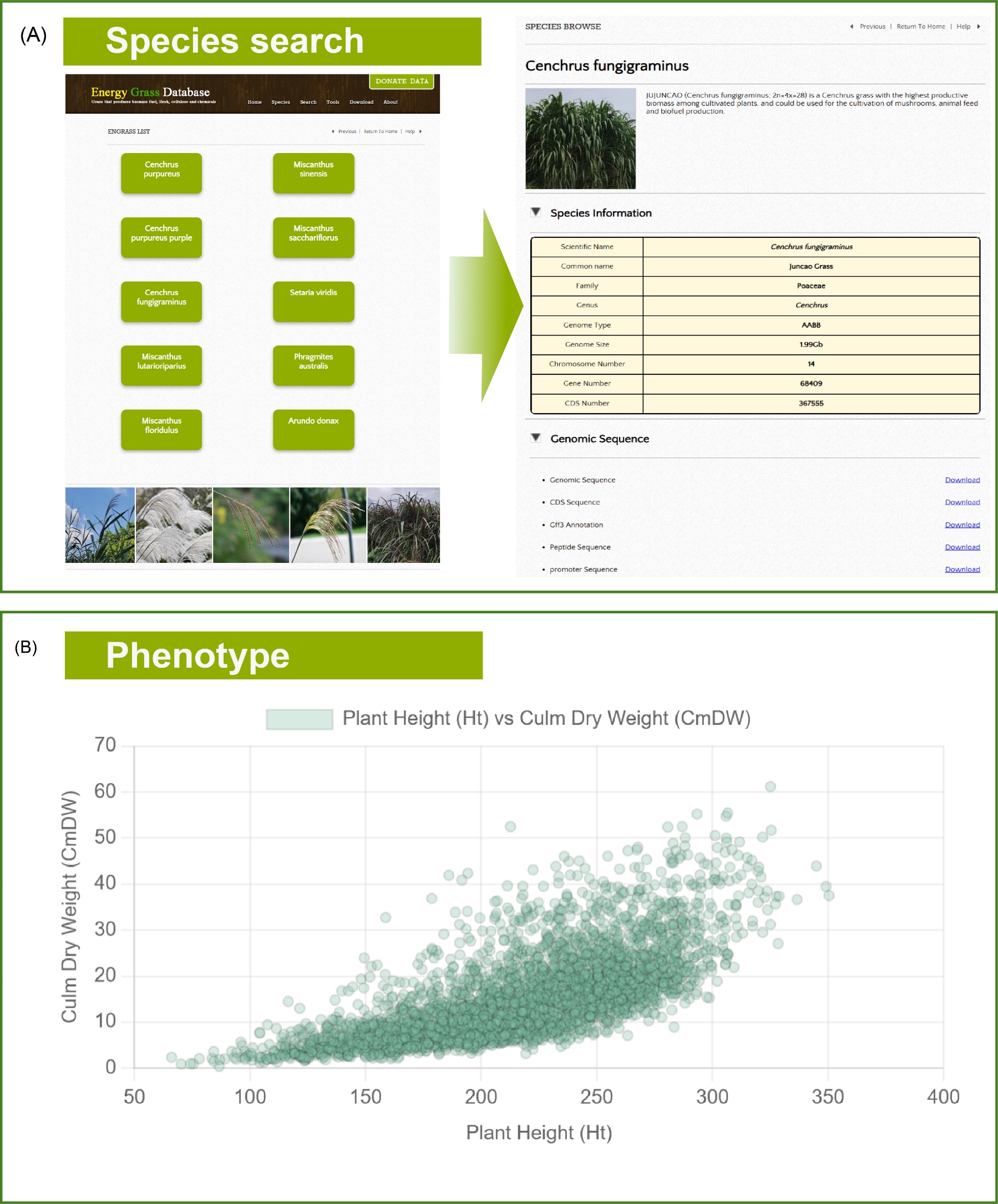


**Figure S2 Content display of the search module in the Energy Grass Database (EGDB).** (A) Species search interface and information display: Users can search for energy grass species using the species search interface. Selecting a species, such as *C. fungigraminus*, displays detailed information including scientific name, common name, origin, and genomic sequence availability. (B) Phenotypic trait analysis. This panel provides a visual analysis of key phenotypic traits within the EGDB. Scatter plot showing a positive correlation between plant height (Ht, cm) and culm dry weight (CmDW, g), indicating that taller plants generally have higher biomass yield, highlighting the role of plant height in biomass production.


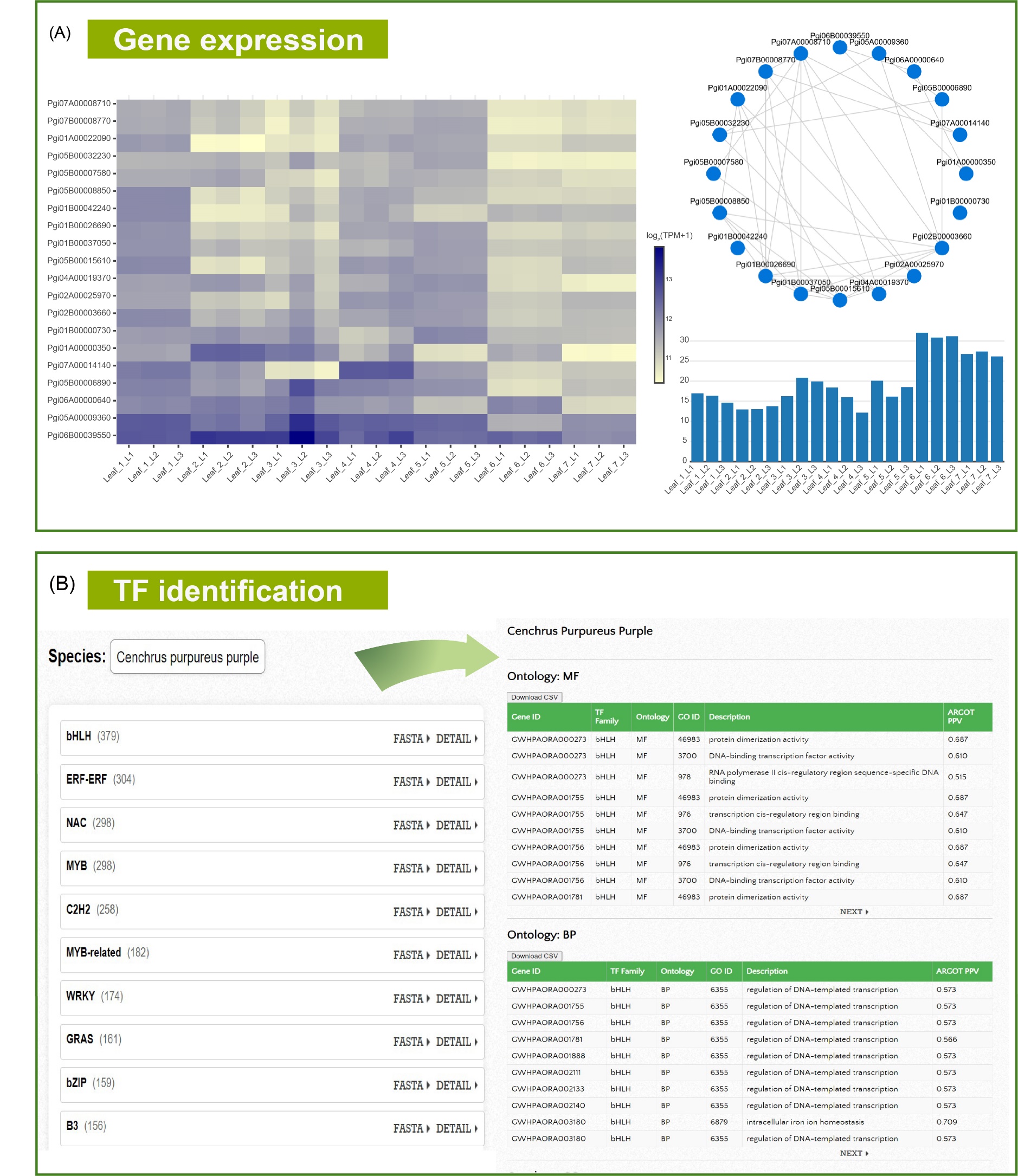


**Figure S3 Gene expression and phenotype analysis functions in the EGDB.** (A) Gene expression analysis. Heatmap of the top 20 highly expressed genes in *C. fungigraminus* across seven developmental stages of leaf growth, encompassing tillering (Leaf_1-3), jointing (Leaf_4-5), and maturity stages (Leaf_6-7), each with three biological replicates (L1-3). Expression values are presented as log2(TPM (Transcripts Per Million) + 1) to enhance interpretability. The color gradient reflects gene expression dynamics, providing insight into expression patterns across developmental stages. Co-expression network of *C. fungigraminus*, derived from the gene expression module. Nodes represent individual genes, while edges indicate gene pairs with a Pearson correlation coefficient ≥ 0.8, indicating strong co-expression. This network aids in identifying potential regulatory interactions between genes during leaf development stages. Expression profile of a specific gene (e.g., *Pgi06A00010030*) queried via the interface, shown as a bar plot across seven developmental stages. The X-axis represents developmental stages, and the Y-axis indicates expression levels in TPM. This plot allows a detailed examination of gene-specific expression changes throughout development. (B) TF identification and ontology data retrieval: The TF identification module enables users to explore transcription factors associated with a selected species. Shown is the ontology-based categorization, including molecular function (MF) and biological process (BP) terms, along with TF-related details such as ID, category, and functional descriptions for easy access.


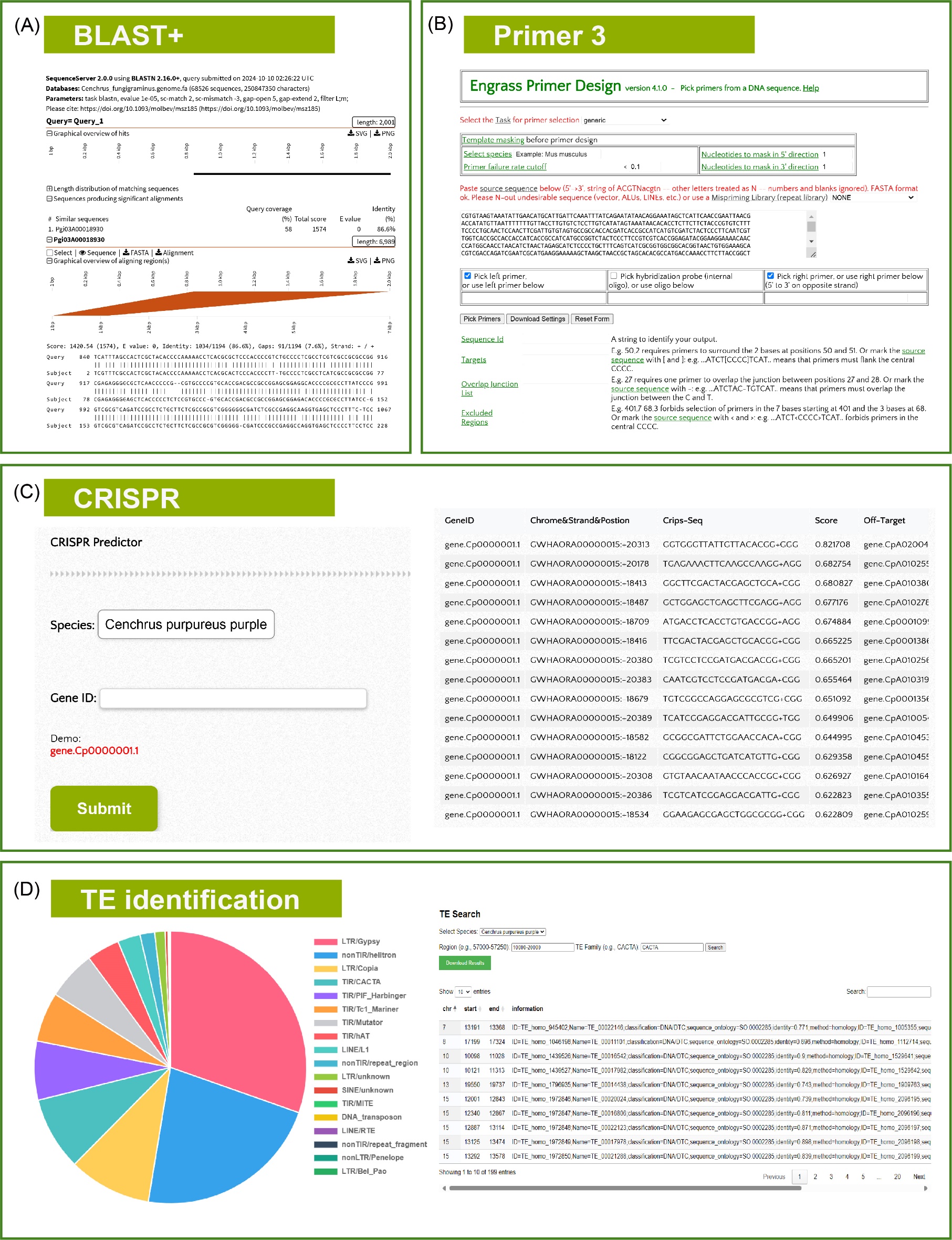


**Figure S4 The tools of the EGDB.** (A) BLAST analysis tool: This figure displays results obtained from BLAST analysis using a promoter sequence , enabling researchers to identify similar sequence for further functional analysis in energy grass genomes. (B) PRIMER design tool: This figure illustrates the primer design feature, allowing efficient primer design for various molecular biology applications in grass species studies. (C) CRISPR guide RNA design tool: This figure shows the CRISPR guide RNA design function using the *gene.cp000000.1* of *C.purpureus* cv. Purple as an example. Upon submission, users receive potential guide RNA sequences for targeted genome editing in grass species. (D) TE identification overview: a pie chart (left) displaying the proportional distribution of different TE families, with LTR/Gypsy representing the largest proportion, followed by helitron and LTR/Copia, among others. The interactive search interface (right) allows users to filter and query TEs in *C. purpureus* cv. Purple by genomic region and TE families. Results include chromosome, start and end positions, TE details, reading phase, DNA strand, and TE families. The interface supports pagination and a CSV download option for exporting filtered results for further analysis.


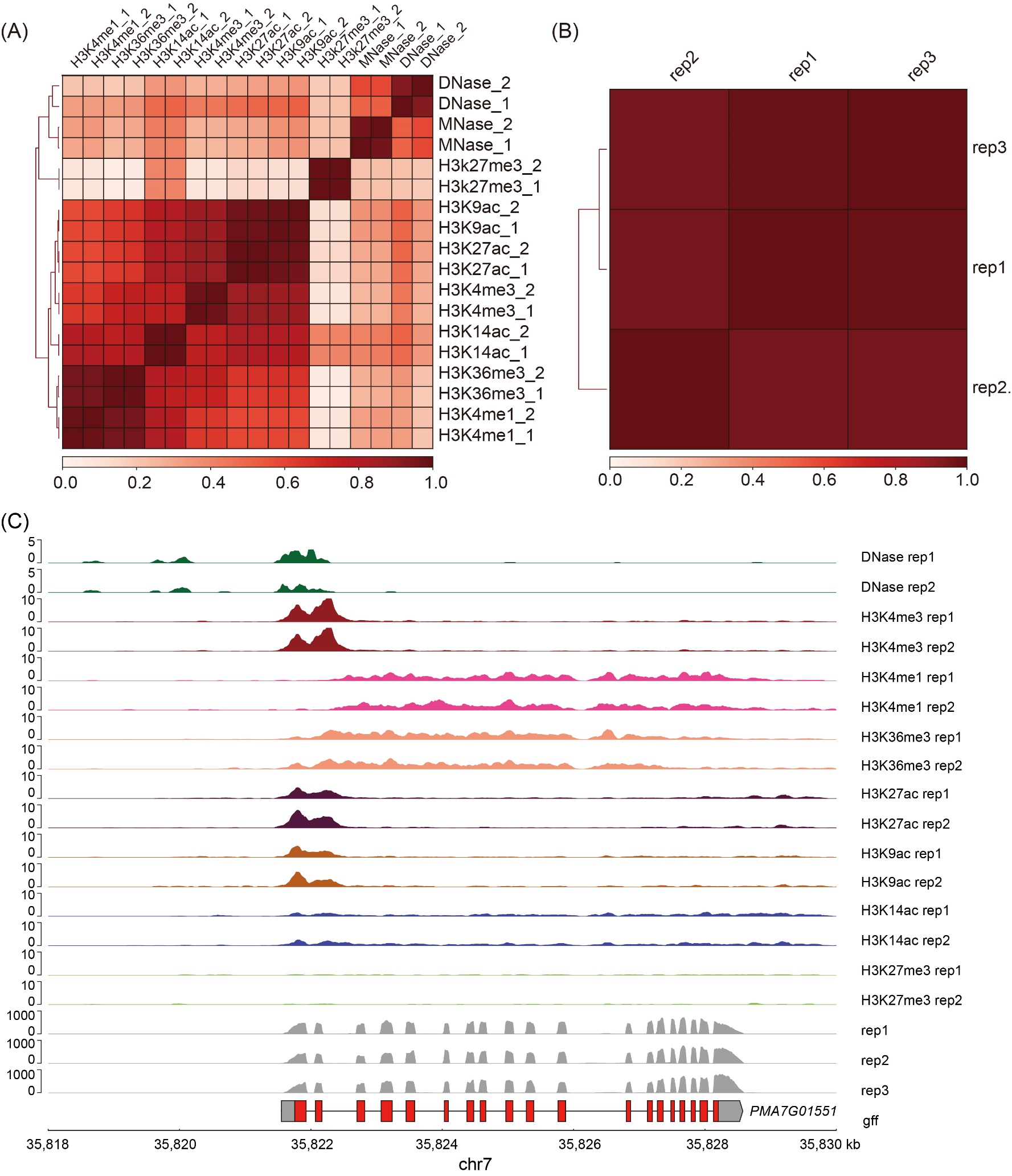


**Figure S5 Epigenomic marks featured by ChIP-seq.** (A) Heatmap showing high reproducibility between biological replicates of ChIP-seq data for various histone modifications and DNase hypersensitivity in pearl millet. (B) Heatmap indicating a strong correlation between biological replicates in RNA-seq data. (C) Genome browser view of ChIP-seq tracks for a young leaf sample of pearl millet, displaying data from two biological replicates for various histone modifications and DNase hypersensitivity near *PMA7G01551.*

**
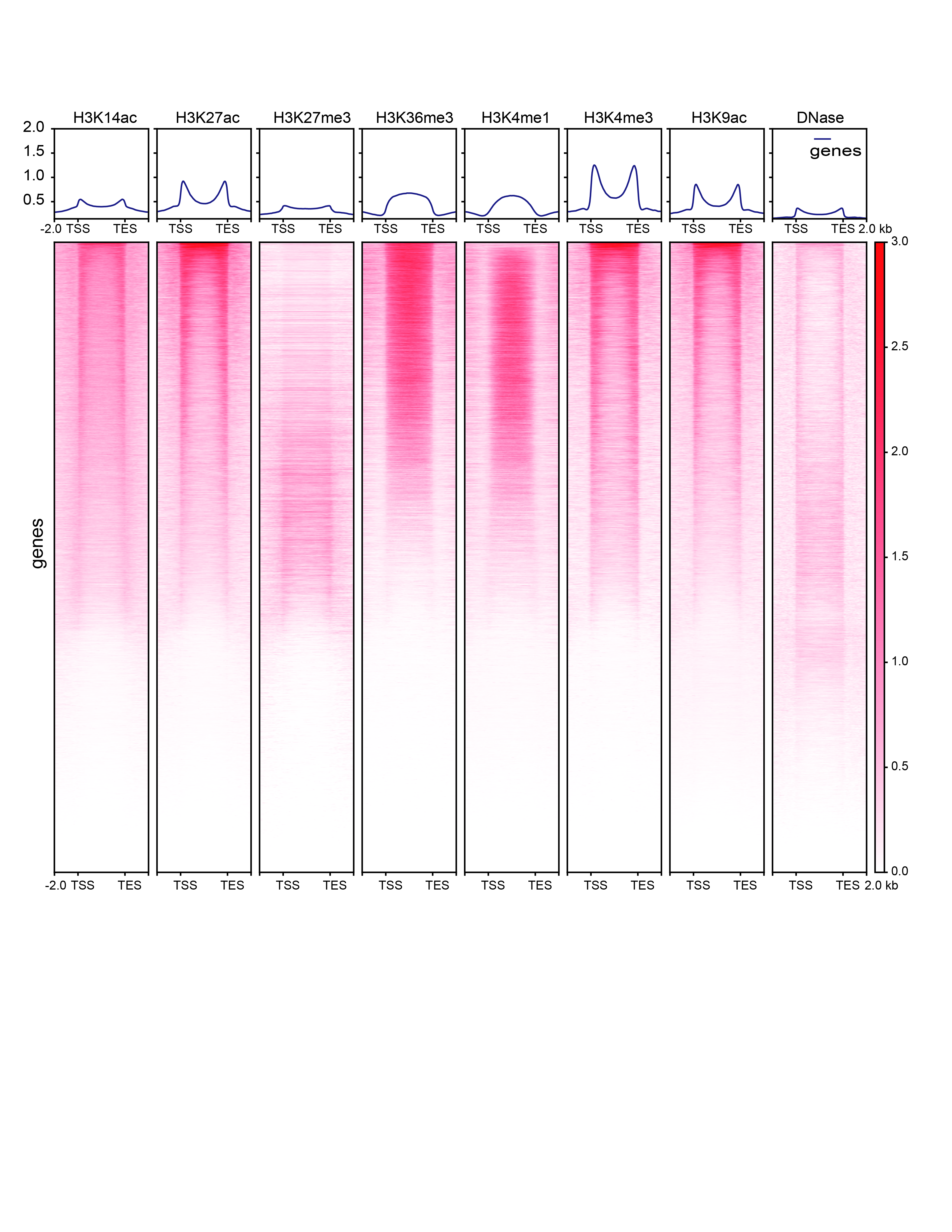
**

**Figure S6** **Distribution of modification level of seven histone modifications and DHS across all genes in the genome of pearl millet.** Each panel shows the average signal intensity from 2 kb upstream of the transcription start site (TSS) to 2 kb downstream of the transcription end site (TES).


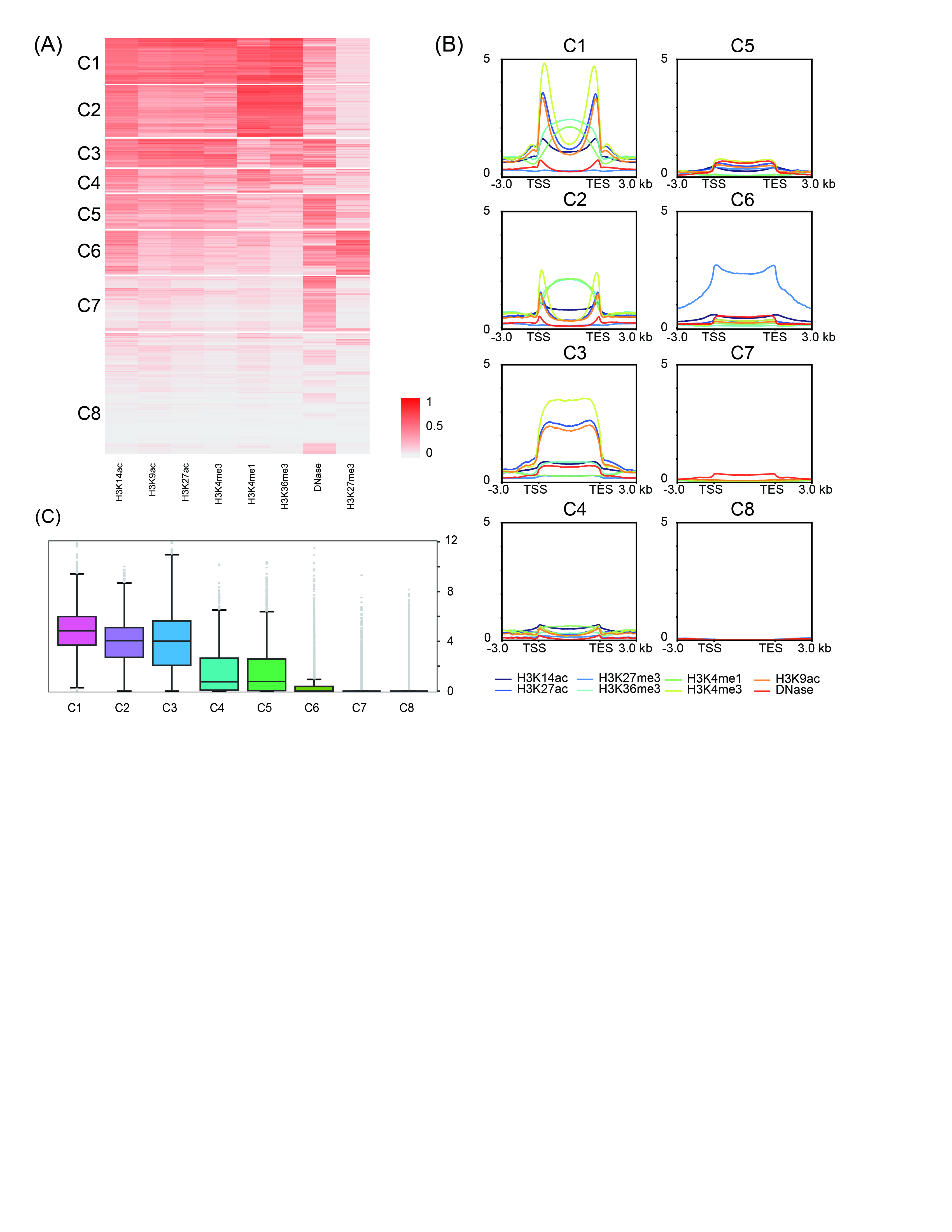


**Figure S7** **Clustering analysis reveals that genes associated with histone modifications exhibit varying levels of expression.** (A) Eight gene clusters defined by combinations of seven epigenomic marks and DNase hypersensitivity site (DHS) density. (B) Distribution of peaks for each of the seven epigenomic marks and DHS density around genes in each cluster. (C) Gene expression levels across the eight clusters.


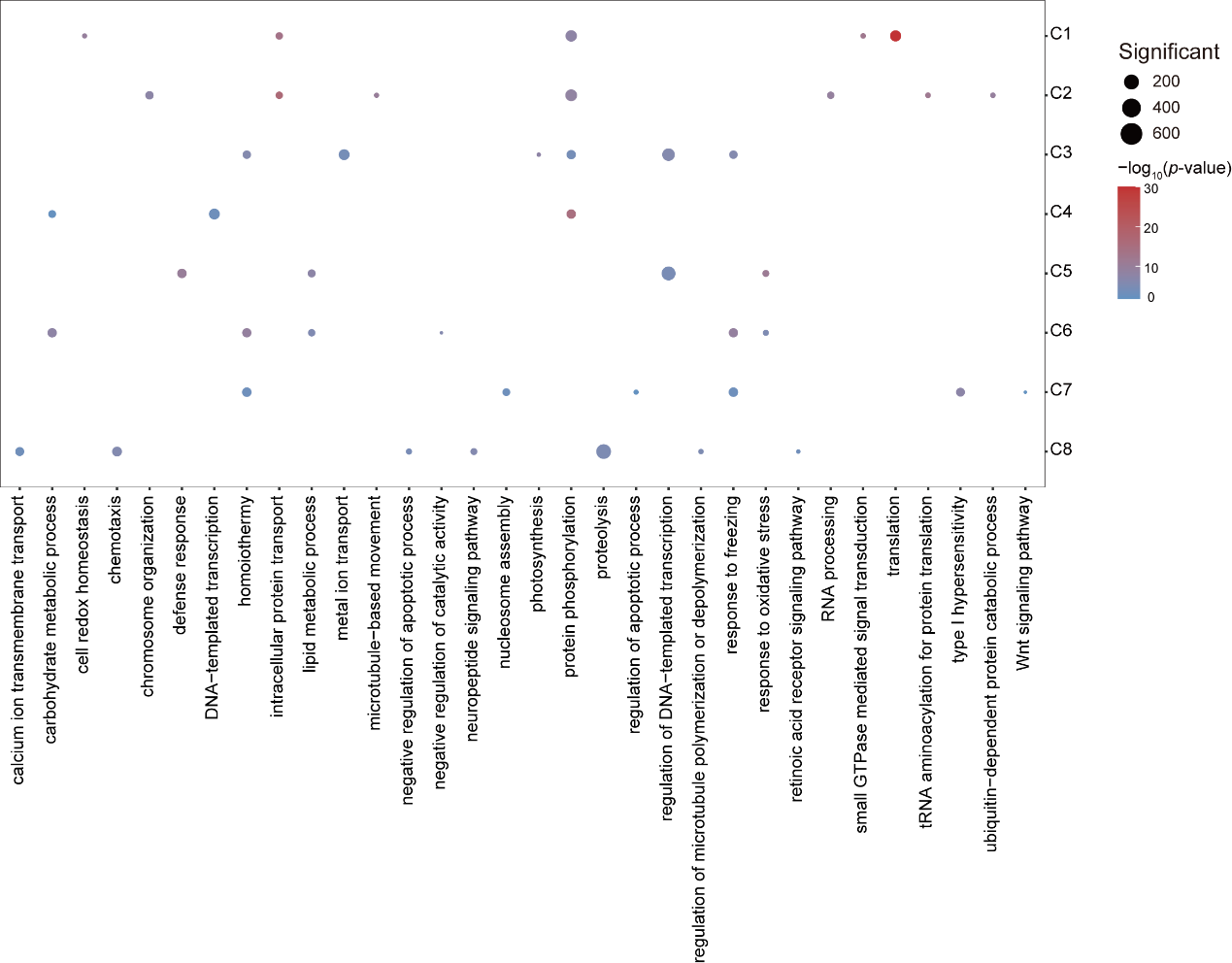
**Figure S8 GO enrichment analysis was performed on the genes in the eight clusters.** These genes were grouped into eight clusters based on the epigenomic features of their surrounding regions and promoters. The significantly enriched biological process terms for each cluster are displayed in the plot.


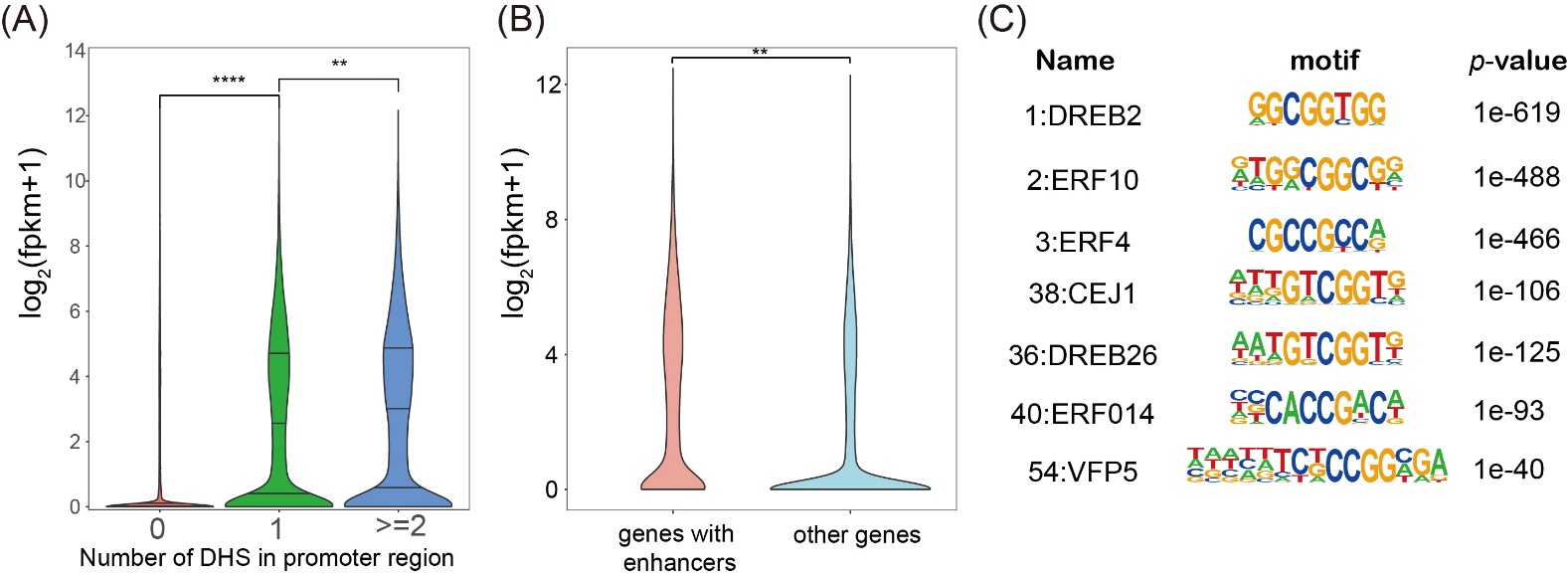


**Figure S9 The architecture of open chromatin region in pearl millet.** (A) Correlation between open chromatin regions and gene expression levels. (B) Genes with identified enhancers show significantly higher expression levels compared to genes without enhancers. (C) Enriched motifs of transcription factor binding site (TFBS) footprints.


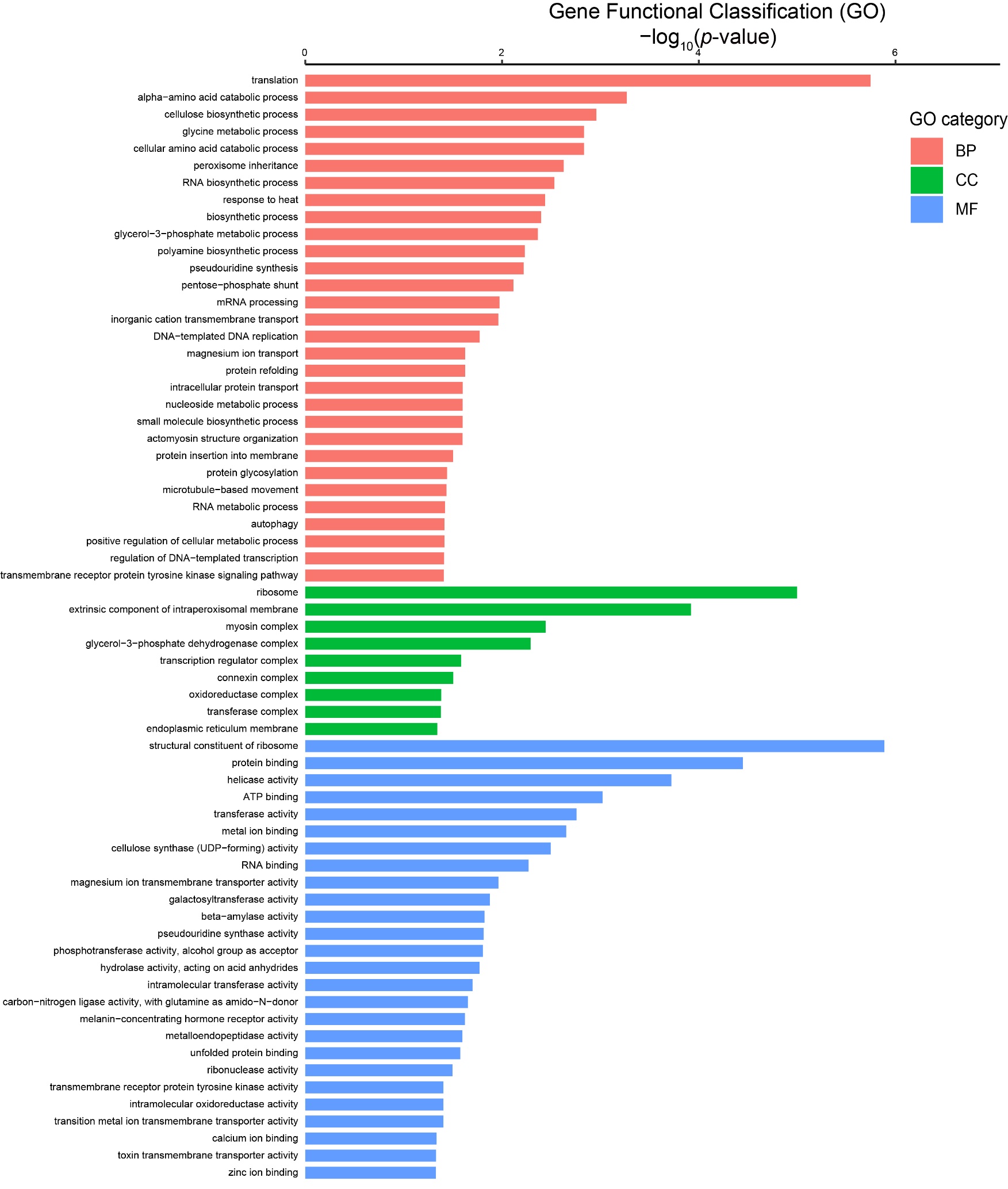


**Figure S10 GO enrichment analysis of genes targeted by AP2 transcription factors.** Those genes were identified based on transcription factor binding sites (TFBS). The significantly enriched biological processes (BP), molecular functions (MF), and cellular components (CC) for each cluster are displayed in the plot.


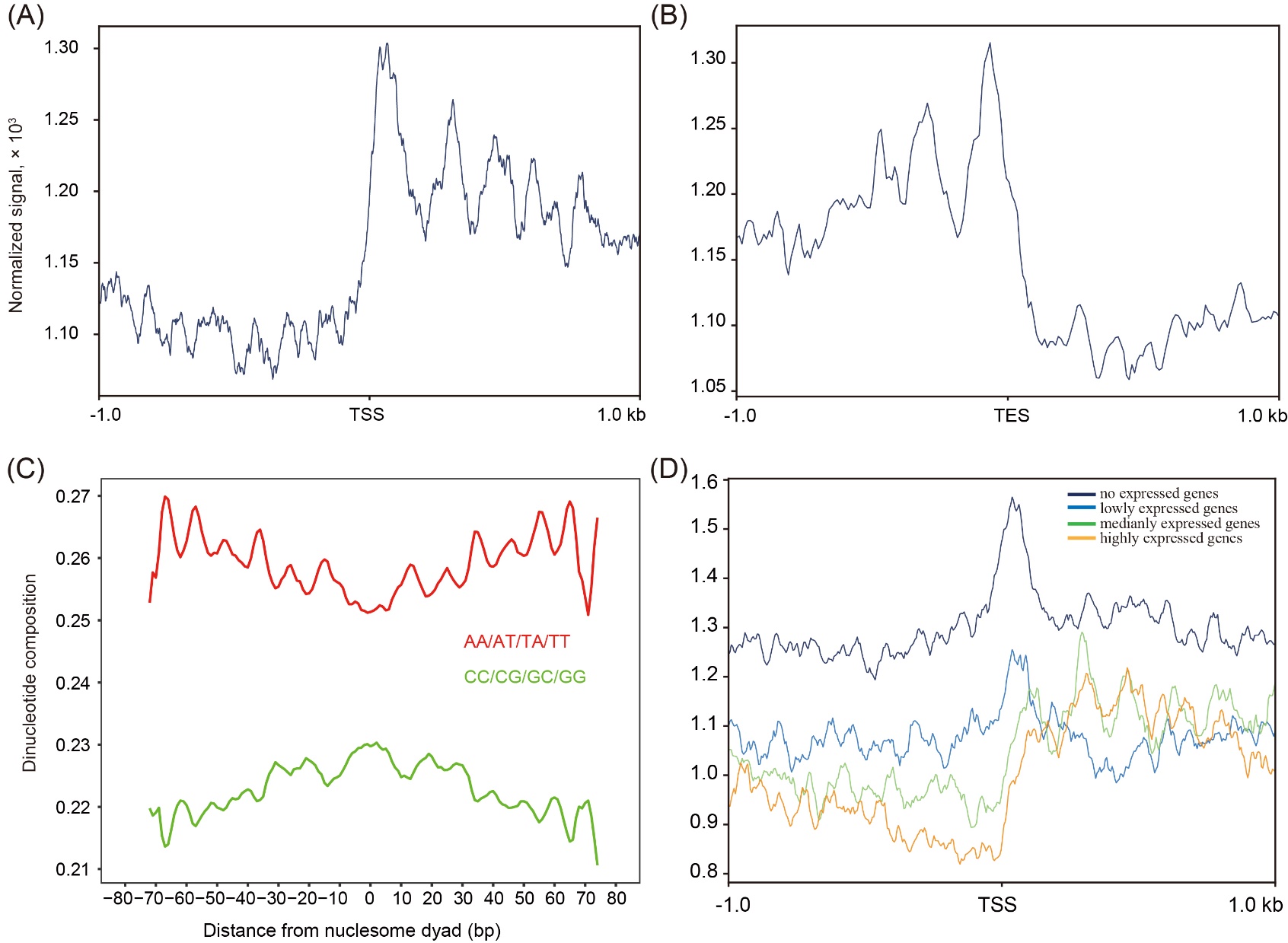


**Figure S11 The landscape of nucleosome position and occupancy in pearl millet.** (A−B) Average nucleosome occupancy patterns near transcription start sites (TSS) and transcription end sites (TES) of all genes. (C) Composite distribution of AA/AT/TA/TT and CC/CG/GC/GG dinucleotides in nucleosomal DNA. (D) Average nucleosome occupancy patterns near the TSS of genes grouped by four different levels of expression.


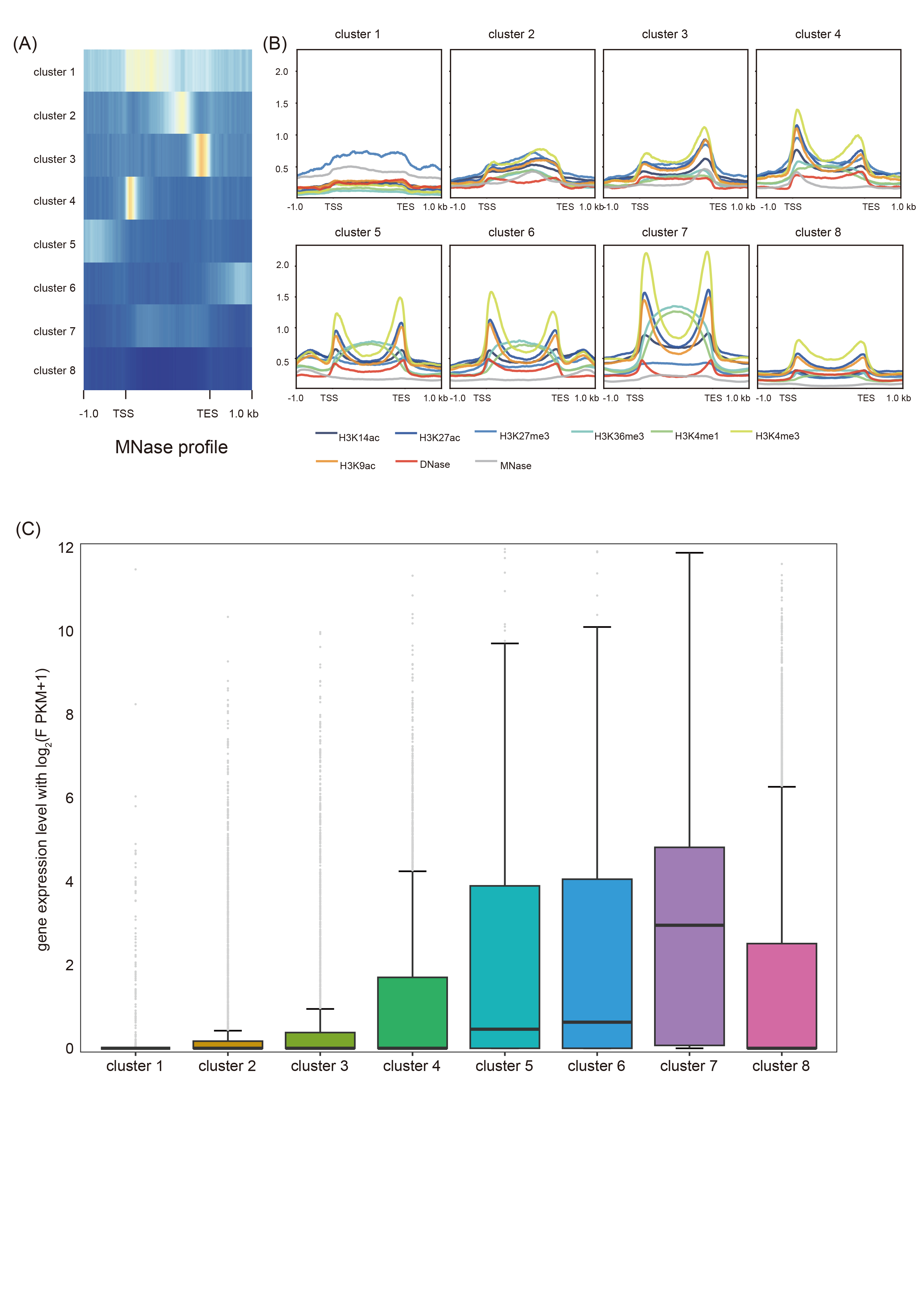


**Figure S12 The epigenetic and transcriptomic patterns of genes based on their corresponding nucleosome occupancy.** The eight nucleosome occupancy patterns were identified in gene regions (A), associated epigenomic features (B), and corresponding gene expression levels (C).


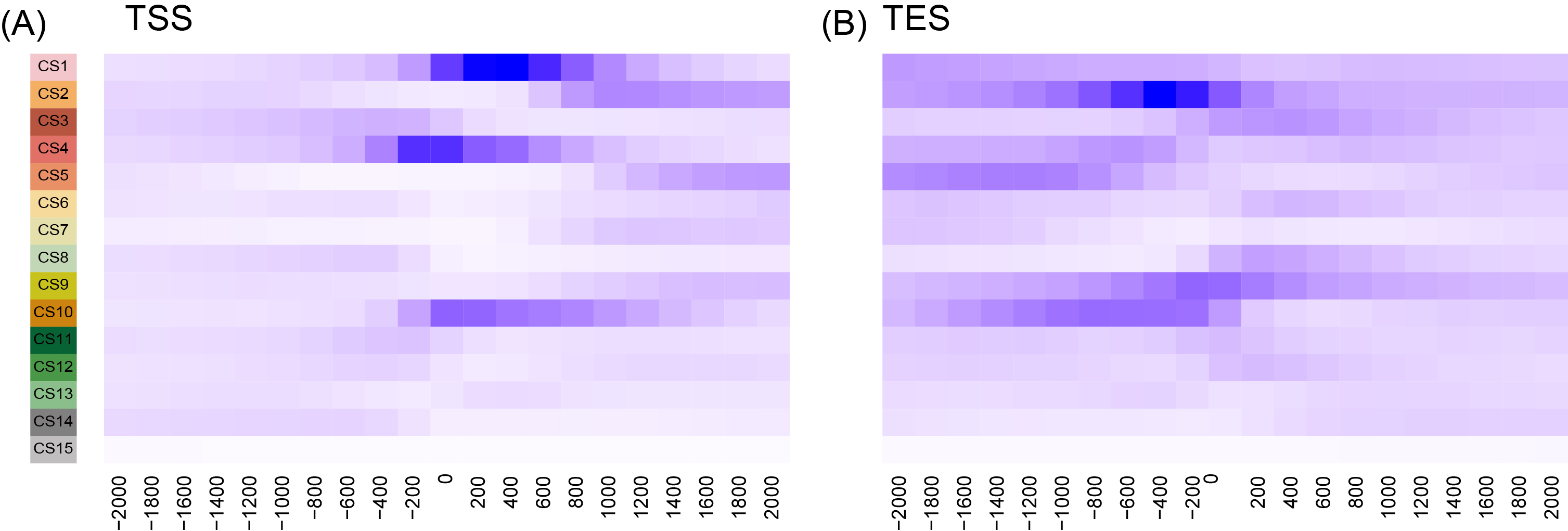


**Figure S13 Chromatin State Distribution around TSS and TES.** Distribution of 15 chromatin states around the transcription start site (TSS) (A) and transcription end site (TES) (B) of genes.

**
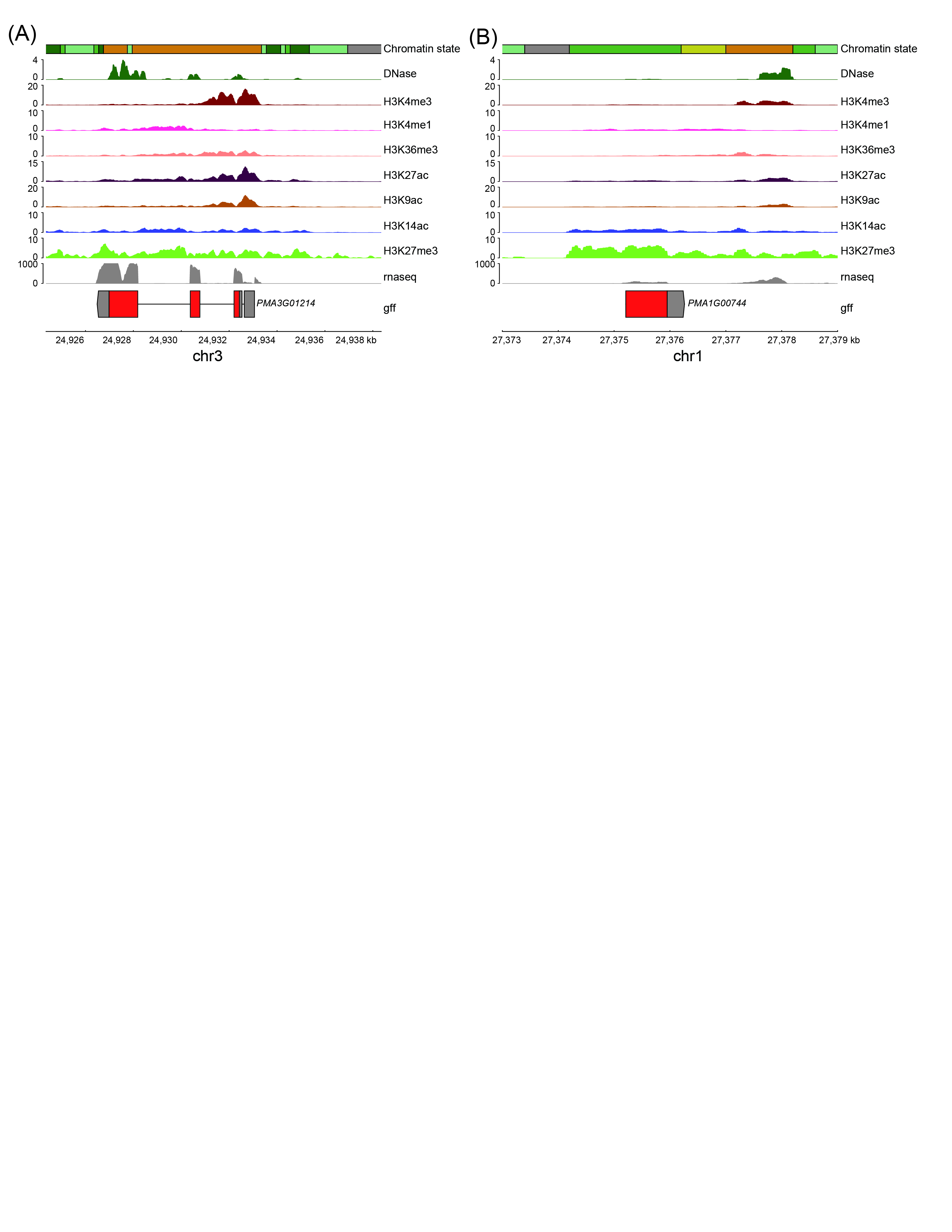
**

**Figure S14 Examples of genes affected by two newly identified chromatin states.** (A) *PMA3G01214* is associated with chromatin state CS11/ReprInt. (B) *PMA1G00744* is associated with chromatin state CS12/EnhBiv. Examples of genes affected by two newly identified chromatin states.


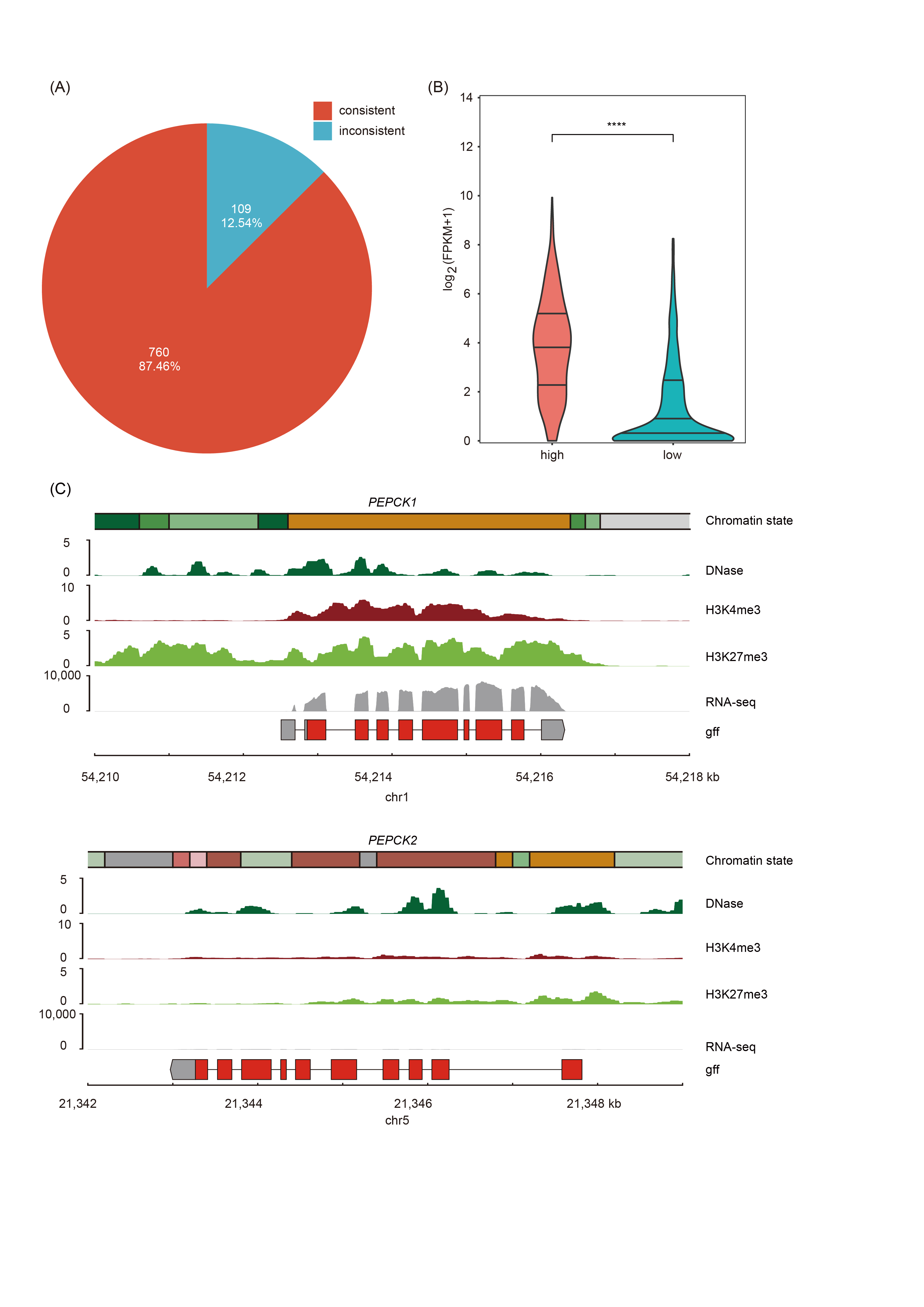


**Figure S15** **Imbalanced epigenetic modifications contribute to differential gene expression.** (A) Proportion of duplicated orthologues with consistent (87.46%) and inconsistent (12.54%) expression patterns relative to H3K4me3 levels. (B) Expression comparison of duplicated paralogues: copies with higher H3K4me3 levels (high) exhibit significantly greater expression than those with lower H3K4me3 (low) (**** indicates significance). (C) Epigenetic profiles of duplicated PEPCK genes, where *PEPCK1* is marked with both H3K4me3 and H3K27me3 (top), and *PEPCK2* is marked only with H3K27me3 (bottom).


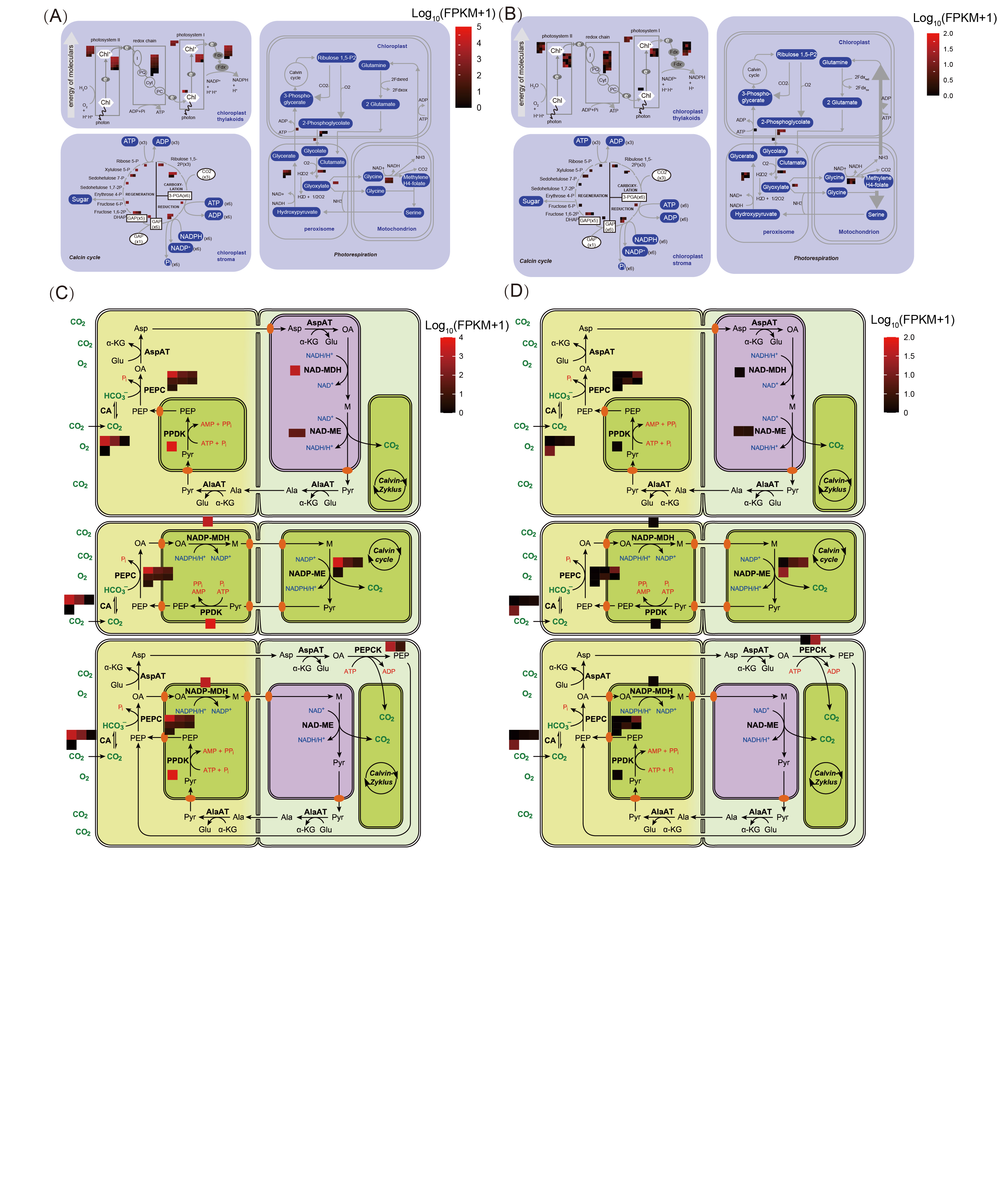


**Figure S16 Epigenetic and transcriptomic profiles in pearl millet.** Expression profile and H3K4me3 intensity of genes involved in the “Photosynthesis” pathway (A−B) and the “Photosynthesis_C4 photosynthesis” pathway (C−D), as mapped by MAPMAN.
